# Gene expression analysis of human induced pluripotent stem cell-derived neurons carrying copy number variants of chromosome 15q11-q13.1

**DOI:** 10.1186/2040-2392-5-44

**Published:** 2014-08-20

**Authors:** Noelle D Germain, Pin-Fang Chen, Alex M Plocik, Heather Glatt-Deeley, Judith Brown, James J Fink, Kaitlyn A Bolduc, Tiwanna M Robinson, Eric S Levine, Lawrence T Reiter, Brenton R Graveley, Marc Lalande, Stormy J Chamberlain

**Affiliations:** 1 Department of Genetics and Developmental Biology, University of Connecticut Health Center, 400 Farmington Avenue, Farmington, CT 06032, USA; 2 Chromosome Core, Department of Molecular and Cell Biology and Department of Allied Health Sciences, University of Connecticut, 354 Mansfield Road, Storrs, CT 06269, USA; 3 Department of Neuroscience, University of Connecticut Health Center, 263 Farmington Avenue, Farmington, CT 06030, USA; 4 Department of Neurology, University of Tennessee Health Science Center, 855 Monroe Avenue, Suite 415, Memphis, TN 38163, USA; 5 University of Connecticut Institute for Systems Genomics, Farmington, CT 06030, USA

**Keywords:** UBE3A, autism, induced pluripotent stem cells, 15q duplication, Angelman syndrome

## Abstract

**Background:**

Duplications of the chromosome 15q11-q13.1 region are associated with an estimated 1 to 3% of all autism cases, making this copy number variation (CNV) one of the most frequent chromosome abnormalities associated with autism spectrum disorder (ASD). Several genes located within the 15q11-q13.1 duplication region including ubiquitin protein ligase E3A (*UBE3A*), the gene disrupted in Angelman syndrome (AS), are involved in neural function and may play important roles in the neurobehavioral phenotypes associated with chromosome 15q11-q13.1 duplication (Dup15q) syndrome.

**Methods:**

We have generated induced pluripotent stem cell (iPSC) lines from five different individuals containing CNVs of 15q11-q13.1. The iPSC lines were differentiated into mature, functional neurons. Gene expression across the 15q11-q13.1 locus was compared among the five iPSC lines and corresponding iPSC-derived neurons using quantitative reverse transcription PCR (qRT-PCR). Genome-wide gene expression was compared between neurons derived from three iPSC lines using mRNA-Seq.

**Results:**

Analysis of 15q11-q13.1 gene expression in neurons derived from Dup15q iPSCs reveals that gene copy number does not consistently predict expression levels in cells with interstitial duplications of 15q11-q13.1. mRNA-Seq experiments show that there is substantial overlap in the genes differentially expressed between 15q11-q13.1 deletion and duplication neurons, Finally, we demonstrate that *UBE3A* transcripts can be pharmacologically rescued to normal levels in iPSC-derived neurons with a 15q11-q13.1 duplication.

**Conclusions:**

Chromatin structure may influence gene expression across the 15q11-q13.1 region in neurons. Genome-wide analyses suggest that common neuronal pathways may be disrupted in both the Angelman and Dup15q syndromes. These data demonstrate that our disease-specific stem cell models provide a new tool to decipher the underlying cellular and genetic disease mechanisms of ASD and may also offer a pathway to novel therapeutic intervention in Dup15q syndrome.

## Background

One of the most frequent chromosome anomalies associated with autism is the duplication of chromosome 15q11-q13.1 [1-8]. The parent-of-origin is an important factor for chromosome 15q11-q13.1 duplication (Dup15q) syndrome because the chromosome 15q11-q13.1 region is subject to genomic imprinting, which is an epigenetic process that results in monoallelic gene expression. The 15q11-q13.1 duplications that lead to autism are most frequently of maternal origin. In addition to autism, individuals with maternally-inherited or derived duplications of chromosome 15q11-q13.1 have hypotonia, developmental delay, speech and language delay, behavioral difficulties, and seizures. There are two major classes of chromosomal duplication. First, interstitial duplications (int dup(15)) result in tandem copies of maternal 15q11-q13.1 lying in a head-to-head orientation on the same chromosome arm. Second, isodicentric chromosome 15 (idic(15)) duplications result in two additional copies of maternal 15q11-q13.1 which are flanked by two centromeres on a supernumerary chromosome. Not surprisingly, individuals with idic(15), who have 4 copies of 15q11-q13.1, are more severely affected than those with int dup(15), who have 3 copies [9].

Deletion of the maternal allele of chromosome 15q11-q13.1 results in Angelman syndrome (AS) a neurodevelopmental disorder characterized by developmental delay, absent speech and seizures [10]. Deletion or loss of function of a single maternally expressed gene within 15q11-q13.1, encoding ubiquitin protein ligase E3A (*UBE3A*), is sufficient to cause AS [11-13]. Several studies suggest that maternal duplication of *UBE3A* underlies the autism phenotype associated with 15q11-q13.1 duplications [1,2,8,14]. However, the duplicated region also includes several other genes, including a cluster of genes encoding gamma aminobutyric acid (GABA) receptor subunits, *GABRB3*, *GABRG3*, and *GABRA5*. Additionally, all idic(15) duplications and some int dup(15) duplications include cytoplasmic FMRP interacting protein (*CYFIP1*), which binds to and antagonizes the fragile X mental retardation protein (FMRP) [15], the protein whose loss of function causes Fragile X syndrome; and nonimprinted in Prader*-*Willi*/* Angelman syndrome region 1 and 2 (*NIPA1* and *NIPA2*), genes encoding putative magnesium transporters involved in seizures, schizophrenia, and hereditary spastic paraplegia [16,17]. Therefore, non-imprinted genes in the 15q critical duplication locus may play an important role in the phenotype of individuals with both int dup (15) and idic(15).

Here we report the generation of induced pluripotent stem cell (iPSC) lines and neurons from individuals with both isodicentric and interstitial duplications of chromosome 15q11-q13.1. We compared gene expression between iPSCs and iPSC-derived neurons with both deletions and duplications of this region. We found that while the overall gene expression levels of the chromosome 15q genes largely reflect the copy number in AS and idic(15) iPSCs and neurons, the gene expression levels did not correlate as well with copy number in the paternal or maternal int dup(15) neurons, suggesting that the inverted duplication may disrupt distal regulatory elements that act primarily in neural tissue. We also compared global transcriptome expression between AS and idic(15) neurons and found that despite having opposite genetic anomalies (deletion in AS and duplication in idic(15)), most of the genes differentially expressed in both disorders were changed in the same direction. In fact, both disorders result in the downregulation of genes involved in neuron development, including many autism candidate genes. Together, these data suggest different patterns of neuronal gene regulation between int dup(15) and idic(15) and similar neuronal pathways disrupted in deletions and duplication of chromosome 15q11-q13.1.

## Methods

### Patient samples, regulatory approvals, iPSC derivation, and cell culture

Idic(15) fibroblasts (catalog ID: GM07992) were obtained from the Coriell Institute for Medical Research Cell Repository. Idic(15) umbilical cord blood cells were donated by the patient’s family through the Dup15q Alliance and were exempted from consideration as human subject research by the University of Connecticut Health Center Institutional Review Board (IRB). Fibroblasts from an individual with inherited paternal interstitial 15q11-q13.1 duplication (patient 801-015) and an individual mosaic for maternal interstitial 15q11-q13.1 duplication (mother of patient 801-018) were obtained by Dr. Lawrence T. Reiter under IRB approval number 11-01350-FB from the University of Tennessee Health Science Center. All patient samples were obtained from subjects after they had given informed consent and were subsequently de-identified.

iPSCs were generated with Institutional Biosafety Committee approval number IBC08-005 from the University of Connecticut Health Center by the University of Connecticut - Wesleyan University Stem Cell Core as previously reported [18]. iPSCs were maintained on irradiated mouse embryonic fibroblasts (MEFs) in human embryonic stem cell medium which consists of DMEM/F12, 20% knockout serum replacement, 1 mM L-glutamine, 1X nonessential amino acids, 100 mM β-mercaptoethanol (all Gibco products through Life Technologies, Grand Island, NY, USA), and 4 ng/mL basic fibroblast growth factor (bFGF, Millipore, Billerica, MA, USA). iPSCs were manually passaged every 6 or 7 days.

### Karyotype analysis, DNA fluorescence *in situ* hybridization, and whole genome copy number variation analysis

Cytogenetic analysis of Dup15q iPSCs was performed by the Genetics and Genomics Division of the University of Connecticut - Wesleyan University Stem Cell Core. Twenty G-banded metaphase cells from each iPSC line were examined to generate a karyotype for each line. DNA fluorescence *in situ* hybridization (FISH) was performed on both metaphase and interphase cells using a dual-labeled probe containing the small nuclear ribonucleoprotein polypeptide N (*SNRPN*) gene and a control locus at 15qter (Cytocell Aquarius LPU005-A-034359, Cytocell, Cambridge, UK). Whole genome copy number analysis was performed on genomic DNA isolated from AS del 1-0 and Idic1-8 iPSCs using the Affymetrix CytoScan HD Array (Affymetrix, Santa Clara, CA, USA) to determine deletion/duplication breakpoints. Duplication breakpoints for the maternal and paternal Int dup(15) patient samples were previously published (as patients 801-018 and 801-015, respectively) [8] and for IdicCB were provided in an array CGH report accompanying the patient sample.

### Neural differentiation

iPSC-derived neural progenitors were generated by either embryoid body (EB)-based or monolayer differentiation according to established protocols [19,20] with minor modifications. For EB differentiation, iPSC colonies were manually detached from MEF feeders and put into suspension culture rather than enzymatic dissociation. After three weeks of neural differentiation (in both protocols), neural progenitors were plated on poly-ornithine/laminin coated substrates in neural differentiation medium consisting of Neurobasal Medium, B-27 supplement, nonessential amino acids, and L-glutamine (all Gibco products through Life Technologies, Grand Island, NY, USA) supplemented with 1 μM ascorbic acid, 200 μM cyclic adenosine monophosphate (cAMP), 10 ng/mL brain-derived neurotrophic factor (BDNF, Peprotech, Rocky Hill, NJ, USA), and 10 ng/mL glial-derived neurotrophic factor (GDNF, Peprotech, Rocky Hill, NJ, USA). All experiments were conducted on neural cultures that were at least 10 weeks old.

For mithramycin experiments, mithramycin A (Sigma-Aldrich, St. Louis, MO, USA) was prepared as a 1 mM concentrated stock in dimethyl sulfoxide (DMSO, Sigma-Aldrich, St. Louis, MO, USA). Working stocks were diluted in DMSO and added to neural differentiation medium immediately before addition to neural cultures.

### Immunocytochemistry

Immunocytochemistry was performed as previously described [18] on iPSCs or 10-week old iPSC-derived neurons that had been cultured on glass coverslips. The following antibodies and concentrations were used: mouse anti-Tra-1-60 (1:200, Santa Cruz Biotechnology, Inc, Dallas, TX, USA), Nanog (1:200, Abcam, Cambridge, UK), mouse anti-SSEA-4 (1:20, Developmental Studies Hybridoma Bank, Iowa City, IA, USA), chicken anti-MAP2 (1:10,000, Abcam, Cambridge, UK), rabbit anti-MAP2 (1:500, Millipore, Billerica, MA, USA), rabbit anti-Synapsin I (1:400, Millipore, Billerica, MA, USA), mouse anti-PSD-95 (1:100, NeuroMab, Davis, CA, USA), rabbit anti-S100β (1:200, Abcam, Cambridge, UK), mouse anti-VGlut1 (1:100, Synaptic Systems, Gottingen, Germany), and rabbit anti-Gad65 (1:500, Sigma-Aldrich, St. Loius, MO, USA). All AlexaFluor fluorochrome conjugated (488, 594, and 647) secondary antibodies (Life Technologies, Grand Island, NY, USA) were used at 1:400. A goat anti-chicken IgY-650 secondary antibody (Abcam, Cambridge, UK) was used at 1:250. Nuclei were counterstained with DAPI and coverslips were mounted on slides with Vectashield (Vector Laboratories, Burlingame, CA, USA). Slides were imaged using a Zeiss Axiovision microscope (Carl Zeiss, Germany) at 20X, 40X, and 63X magnification.

### Chromatin immunoprecipitation

Chromatin immunoprecipitation (ChIP) was performed according to the EZ-Magna ChIP protocol (Millipore, Billerica, MA, USA) according to manufacturer’s instructions with minor modifications. DMSO or mithramycin-treated 10-week old idic(15) neurons were treated with 1% formaldehyde to crosslink DNA/protein. Instead of a two-step cell lysis, cells were lysed once for 15 minutes in an SDS Cell Lysis Buffer (Millipore, Billerica, MA, USA) before sonication. A rabbit polyclonal anti-YY-1 (sc-281, Santa Cruz Biotechnology, Inc, Dallas, TX, USA) was used at 5 μg per immunoprecipitation reaction. For ChIP analysis of Sp1 binding, the following antibodies were used: mouse monoclonal anti-Sp1 (sc-17824, Santa Cruz Biotechnology, Inc, Dallas, TX, USA) and anti-Sp1 (#9389, Cell Signaling Technology, Beverly, MA, USA). Novex Protein A DynaBeads magnetic beads (Life Technologies, Grand Island, NY, USA) were used during immunoprecipitation. Immunoprecipitated DNA was purified by phenol-chloroform extraction and used for quantitative polymerase chain reaction (qPCR) with SYBR-Green reagents (Life Technologies, Grand Island, NY, USA). The following primers were used for qPCR: *UBE3A*-exon1 forward GGC AGA GGT GAA GCG TAA GT, *UBE3A*-exon1 reverse AGA TCC GTG TGT CTC CCA AG, *UBE3A*-upstream forward TCT GTG ACC CGA AAG AAT AAA CC*, UBE3A*-upstream reverse TTC CTC TGC TGG GTA CAC CAA. *Sp1* promoter forward TGC CCG CCT GAT TTC TGA, *Sp1* promoter reverse GGA TAT GCT TGG GCA AAA TCC, *DHFR* promoter forward TCG CCT GCA CAA ATA GGG AC, *DHFR* promoter reverse AGA ACG CGC GGT CAA GTT TG. ChIP was performed with triplicate independent batches of neurons. qPCR was performed in triplicate for each DNA sample and Ct values were used to calculate percent of input. Percent of input values for YY-1 binding were normalized by subtracting the background binding of normal rabbit IgG (Millipore, Billerica, MA, USA). Fold enrichment was then calculated by dividing percent input in mithramycin-treated samples by percent input of DMSO-treated controls.

### Quantitative reverse-transcription PCR

Total RNA was isolated from iPSCs, EBs, or iPSC-derived neurons using RNA-Bee (AMS Biotechnology, Lake Forest, CA, USA) according to the manufacturer’s protocol. cDNA was produced using the High Capacity cDNA Reverse Transcription Kit (Life Technologies, Grand Island, NY, USA).

Analysis of iPSC pluripotency genes was performed on independent cultures of each iPSC line in triplicate with a TaqMan Human Stem Cell Pluripotency Array (Life Technologies, Grand Island, NY, USA). Analysis of multipotency and neural differentiation capacity was performed with a custom TaqMan Gene Signature Array Card (Life Technologies, Grand Island, NY, USA) as previously described [21] on three independent batches of EBs derived from each iPSC line. This custom array includes representative genes from all three germ lineages as well as pluripotency genes. A full list of gene assays included in the custom array is available in Martins-Taylor *et al*. [21]. Ct values for all genes were first normalized to 18S rRNA and then set relative to the housekeeping gene glyceraldehyde-3-phosphate dehydrogenase (*GAPDH*).

Analysis of 15q11-q13.1 genes and selected autism candidate genes in iPSCs and iPSC-derived neurons was performed in duplicate or triplicate from independent cultures. All qPCR assays used were TaqMan Gene Expression Assays (Life Technologies, Grand Island, NY, USA). Ct values for each gene were normalized to the house keeping gene *GAPDH*. Relative expression was quantified as 2^^-ΔΔCt^ using normal (Nml 1-0) iPSCs or normal iPSC-derived neurons as the calibrator sample.

### Methylation analysis

Analysis of methylation at the Prader-Willi syndrome imprinting center (PWS-IC) was performed using the Methyl-Profiler DNA Methylation qPCR Assay kit (SA Biosciences, Valencia, CA, USA) and the EpiTect Methyl qPCR Assay for *SNRPN* (Qiagen, Valencia, CA, USA) as previously described [18].

### Allele-specific single nucleotide polymorphism analysis

The UCSC Genome Browser (http://genome.ucsc.edu/) was used to identify a single nucleotide polymorphism (SNP), rs691, which is located in the last exon of the imprinted in Prader*-*Willi syndrome (*IPW*) gene. Genomic DNA from mat. int dup(15), pat. int dup(15), and idic(15) fibroblasts, and IdicCB-09 iPSCs was used to determine heterozygosity for rs691 by PCR amplification across the SNP followed by sequencing by GENEWIZ. Total RNA was isolated from fibroblasts (mat. int dup(15) and idic(15)), iPSCs, and iPSC-derived neurons (mat. int dup(15)-02, pat. int dup(15)-04, Idic1-8, and IdicCB-09) using RNA-Bee (AMS Biotechnology, Lake Forest, CA, USA) and DNase treated using Turbo DNA-free Kit (Life Technologies, Grand Island, NY, USA). cDNA was generated using the High Capacity cDNA Reverse Transcription Kit (Life Technologies, Grand Island, NY, USA) and used for PCR amplification across rs691 followed by sequencing by GENEWIZ. The following primers were used to amplify across rs691: forward ATG CCC TCC TCT CTT CCA AT and reverse ATA GGG AGG TTC ATT GCA CA.

### Electrophysiology

Individual coverslips were transferred to a recording chamber (room temperature) fixed to the stage of an Olympus BX51WI microscope (Olympus, Tokyo, Japan) fitted with a 40x water-immersion lens. The recording chamber was continuously perfused at 2 ml/min with oxygenated artificial cerebrospinal fluid (125 mM NaCl, 2.5 mM KCl, 1.25 mM NaH2PO4, 1 mM MgCl2-6H2O, 25 mM NaHCO3, 2 mM CaCl2, and 25 mM dextrose). Cells were selected for recording based on neuronal morphology. Whole-cell voltage clamp (holding potential = -70 mV) and current clamp recordings were conducted as previously described [18,22]. Series resistance (Rs) was compensated to 70% or greater at 10 to 100 μs lag. During the course of the experiments, input resistance (Ri) was continuously monitored with 5 mV hyperpolarizing voltage steps (50 ms). Neurons were rejected from analyses if (1) Rs was >25 MOhms at the time of break-in, if (2) Ri changed by >15% during the course of an experiment, or if (3) Ri fell below 100 MOhms.

### Transcriptome analysis

Total RNA was isolated from two biological replicates of 10-week-old neurons derived from AS (AS del 1- 0), normal (Nml 1- 0), and idic(15) (Idic1-8) iPSCs. mRNA-Seq libraries were prepared using 5 μg of total RNA according to manufacturer’s specifications (Illumina, San Diego, CA, USA) using the Paired-end Library kit as described in [23]. Libraries were multiplexed and sequenced on Illumina GAIIx and HiSeq 2000 sequencers (Illumina, San Diego, CA, USA). The reads were aligned to the human genome (hg19) using Bowtie (version 0.12.0-beta1) and Tophat (version 1.3.1) [24]. At least 20 million mapped reads were generated for each sample. Cufflinks (version 0.9.3) was used to quantitate expression levels for all hg19 UCSC genes and transcripts as fragments per kilobase gene model per million base pairs (FPKM) [25]. All raw sequencing data has been deposited in the National Center for Biotechnology Information (NCBI) Sequence Read Archive (http://www.ncbi.nlm.nih.gov/sra) under the accession number SRP044749.

### Copy number analysis

The copy number of *UBE3A* in each iPSC line was analyzed by qPCR using genomic DNA purified from two biological replicates and TaqMan Copy Number Assays (Hs01665678_cn and Hs03908756_cn, Life Technologies, Grand Island, NY, USA). The RNase P Copy Number Reference Assay was used as an endogenous reference gene to allow for quantification of *UBE3A* copy number. Data were analyzed using the CopyCaller v2.0 software from Applied Biosystems (Life Technologies, Grand Island, NY, USA).

### RNA FISH

iPSCs grown on glass coverslips were processed for RNA-FISH as previously described [21] with the following modifications: *UBE3A* BAC probe RP11-1081A4 was labeled by nick translation and 500 ng of labeled probe was used for hybridization.

### Statistical analyses

Electrophysiology data for current-voltage relationship and average frequency and amplitude of synaptic currents are presented as the mean plus or minus the standard error of the mean. All qPCR data were analyzed using Microsoft Excel and are presented as the mean relative expression plus or minus the standard error of the mean. Differential gene expression was analyzed for statistical significance using either one-way ANOVA followed by Tukey’s multiple comparisons test or an unpaired two-tailed *t*-test. ChIP data are presented as the mean fold enrichment plus or minus the standard error of the mean. Differences in fold enrichment were analyzed for statistical significance using an unpaired two-tailed *t*-test.

## Results

### Generation of iPSCs from Dup15q patients

**Figure 1:**
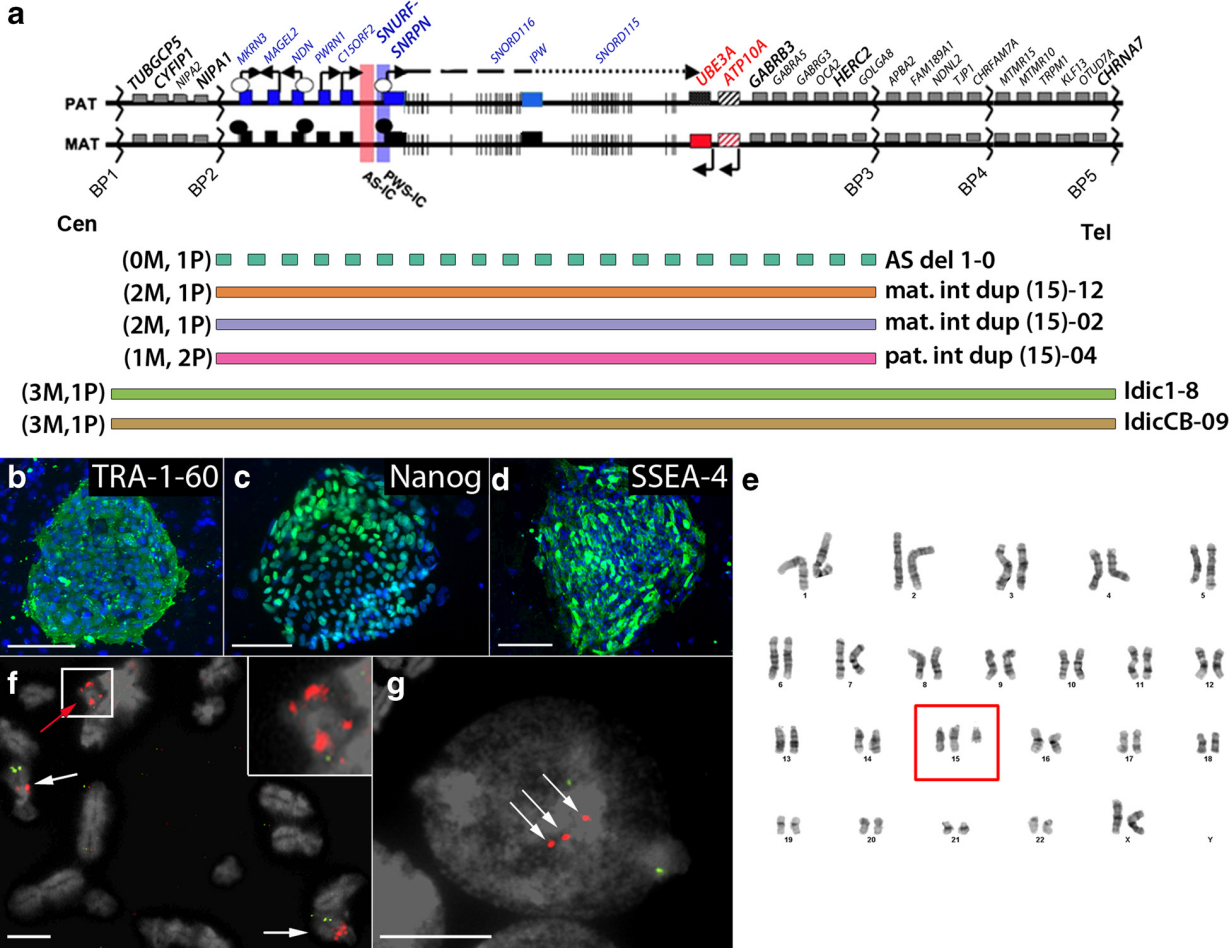
**Characterization of chromosome 15q11-q13.1 duplication***(***Dup15q) induced pluripotent stem cell (iPSCs)*. *(a)** Map of the 15q11-q13.1 region. Boxes indicate genes (grey = biallelically expressed, red = maternally expressed, blue = paternally expressed) and lines indicate small nucleolar RNAs (snoRNAs). The striped boxes for *ATP10A* signify its variable imprinting status. The *UBE3A* anti-sense transcript is represented by the dotted line beginning at *SNURF*-*SNRPN*. Pink and blue shaded regions demarcate the Angelman Syndrome and Prader-Willi Syndrome imprinting centers (AS-IC and PWS-IC), respectively. Open circles represent unmethylated CpG islands, while the corresponding black circles on the maternal allele represent methylated CpG islands in silenced genes (black boxes). Breakpoints (BP1-BP5) are indicated. The regions deleted or duplicated in iPSC lines used in this study as well as their respective maternal (M) or paternal (P) allele copy numbers are indicated below the map. **(b-d)** Immunocytochemistry for the pluripotency markers TRA-1-60, Nanog, and SSEA-4 on idic(15) iPSC colonies (Idic1-8). Nuclei are counterstained with DAPI (blue). **(e)** Karyogram of idic(15) iPSCs shows a karyotype of 47,XX,+idic(15).ish15q12 SNRPN x 4, 15qter X2. Red box indicates the supernumerary isodicentric chromosome 15. **(f, g)** DNA FISH on metaphase idic(15) iPSCs (F) and interphase int dup(15) iPSCs (G) with a probe for *SNRPN* (red) and a control probe on the distal long arm of chromosome 15 (green). Two normal chromosomes 15 are indicated by white arrows (F). The isodicentric chromosome 15 (highlighted in white box and inset) shows four signals for *SNRPN* and no signal for the control probe (F). Three signals for *SNRPN* and two control signals indicate the presence of the interstitial duplication of 15q11-q13.1 on one chromosome 15 (G). Scale bars in (B) and (D) are 200 μm, in (C) is 100 μm, in (F) is 5 μm and in (G) is 10 μm.

iPSC lines were established using fibroblasts from one idic(15) individual, one individual with a paternally-inherited duplication of chromosome 15q11-q13.1, and one individual who was mosaic for a maternally-inherited interstitial duplication of chromosome 15q11-q13.1 (Figure 1a). iPSC lines were also established from a cord blood sample from one individual with idic(15) (Figure 1a). iPSCs were generated using either retroviral, lentiviral, or episomal vectors encoding POU class 5 homeobox 1 (*OCT4*), SRY-box 2 (*SOX2*), Kruppel-like factor 4 (*KLF4*), v-myc avian myelocytomatosis viral oncogene homolog (*MYC*), and lin28 homolog A (*LIN28*) [see Additional file 1: Table S1] [26-28]. Reprogrammed colonies were initially identified morphologically, and were subsequently validated using qRT-PCR and/or immunocytochemistry to verify the expression of the pluripotency markers, NANOG, stage specific embryonic antigen 4 (SSEA4), and TRA1-60 (Figure 1b-d) and pluripotency genes [see Additional file 2: Figure S1A]. iPSCs were shown to have the expected karyotypes of 46, XX (normal); 46,XX.ish dup(15)(q11.2q11.2)(SNRPN++); or 47,XX,+idic(15).ish15q12 SNRPN x 4, 15qter X2 (Figure 1e). Interstitial duplication of 15q11-q13.1 or the presence of the isodicentric chromosome 15 was confirmed in representative iPSC clones by DNA FISH with a probe for the *SNRPN* gene and a control probe in the distal long arm of chromosome 15 (Figure 1f and Figure 1g). Breakpoints involved in the duplications in the two int dup(15) samples were previously identified by array comparative genomic hybridization (CGH) [8]. Breakpoints in the cord blood idic(15) sample were determined by genetic report obtained during diagnosis, and the breakpoints in the fibroblast idic(15) and AS samples were determined using a high density SNP array as part of the current study. Gene expression arrays were used to analyze gene expression in iPSC-derived EBs after 16 days of spontaneous differentiation as previously described [18,21]. These data demonstrate that all iPSCs used are capable of multi-lineage differentiation, since early lineage markers representing each of the three embryonic germ layers as well as the trophectoderm layer are expressed [see Additional file 2: Figure S1B].

### Dup15q iPSCs maintain the appropriate methylation imprint following reprogramming

We previously demonstrated that the methylation imprint at the PWS-IC was maintained during reprogramming in AS, PWS and normal iPSCs [18]. We assessed the methylation imprint at the PWS-IC in normal and Dup15q iPSCs using methylation-specific qPCR [29,30]. We expected that iPSCs from idic(15) iPSCs would have approximately 75% methylation due to the presence of 3 maternal and 1 paternal alleles. We expected that paternal int dup(15) iPSCs would have approximately 33% methylation resulting from one maternal and two paternal alleles. We expected that some of the iPSCs from the individual mosaic for a maternal int dup(15) would arise from cells with the duplication and thus would have 66% methylation resulting from 2 maternal and one paternal alleles, while others would be derived from the normal cells and have 50% methylation. Consistent with this prediction, idic(15) iPSCs had methylation levels ranging from 71% to 84% and paternal int dup(15) iPSCs had methylation levels ranging from 23% to 27% (Table 1, Additional file 3: Table S2). Two iPSC lines from the mosaic int dup(15) individual had methylation levels of 54% and 57% [see Additional file 3: Table S2]. One line showed normal diploid cells by karyotype analysis (mat. int dup(15)-12), while the other was a mixed culture, having both duplication and normal cells (mat. int dup(15)-18). The remaining iPSC clones from the mosaic individual had methylation levels ranging from 65% to 68% (Table 1). Thus, DNA methylation at the PWS-IC is consistent with the predicted copy number variations of the Dup15q cell lines and seems to be maintained during reprogramming.

**Table 1:** **Methylation analysis of chromosome 15q11-q13.1 duplication syndrome (Dup15q) induced pluripotent stem cells (iPSCs) at the Prader**-**Willi syndrome imprinting center (PWS**-**IC)**

**Cell line**	**% methylated at PWS**-**IC**^**a**^
***mat. Int dup***(***15***)	**66.57 ± 0.92**
mat. Int dup(15)-02	66.15 ± 2.12
mat. Int dup(15)-12^b^	65.64 ± 3.25
***pat. Int dup***(***15***)	**25.31 ± 1.46**
pat. Int dup(15)-04	23.53 ± 2.93
***Idic***	**76.94 ± 5.02**
Idic1-8	72.43 ± 1.00
***IdicCB***	**77.35 ± 3.52**
IdicCB-07	79.49 ± 5.03
IdicCB-09	72.29 ± 9.69

^a^DNA methylation at the PWS-IC was assayed by methylation specific qPCR using genomic DNA from Dup15q iPSCs. The average calculated percent methylation from all clones within a genotype is presented plus or minus the standard deviation in bold text. The averages calculated for percent methylation from three replicate assays each for selected clones plus or minus the standard deviation are shown in plain text. ^b^The mat. int dup(15)-12 cell line was originally identified as an isogenic normal clone derived from the individual with maternal interstitial duplication. However, methylation analysis after approximately 25 subsequent passages suggests that this clone was mosaic and resolved to contain predominantly duplication containing cells. PWS-IC, Prader-Willi syndrome imprinting center; mat. Int dup(15), maternal interstitial duplication of 15q11-q13.1; pat. Int dup(15), paternal interstitial duplication of 15q11-q13.1; Idic, isodicentric chromosome 15; IdicCB, isodicentric chromosome 15 cord blood derived sample.

To further investigate the maintenance of parental imprinting following reprogramming of patient cells into iPSCs and differentiation of iPSCs into neurons, we performed allele-specific RT-PCR for the paternally expressed imprinted in Prader*-*Willi syndrome (*IPW*) gene. We first identified a SNP (rs691) in the last exon of *IPW* and confirmed its heterozygosity in genomic DNA from the mat. int dup(15), pat. int dup(15), and idic(15) patient fibroblasts [see Additional file 4: Figure S2]. We were unable to investigate the SNP in genomic DNA from the umbilical cord blood idic(15) starting cell population; however, we did confirm heterozygosity in genomic DNA from the iPSCs derived from these cells. We then generated cDNA from the patient fibroblasts, iPSCs, and neural derivatives and, by sequencing across the SNP in *IPW*, confirmed that *IPW* is expressed monoallelically and from the same allele as in the patient cell sample [see Additional file 4: Figure S2]. Together with the DNA methylation pattern at the PWS-IC, this data suggests that parental imprinting at the 15q11-q13.1 locus is maintained following reprogramming to iPSCs.

### Functional neurons derived from Dup15q iPSCs

To prioritize individual iPSC lines based on their ability to differentiate into neurons, all clones of the Dup15q iPSCs from each genotype were allowed to spontaneously differentiate in EB culture and were subjected to qRT-PCR using a custom PCR array containing a panel of neural genes and pluripotency genes [21]. Expression levels of all genes in the array were analyzed relative to those of EBs derived from AS iPSCs, which we previously reported to be capable of generating functional neurons [see Additional file 5: Figure S3A-D] [18]. Representative iPSC clones of each genotype were selected for future experiments based on low levels of expression of the pluripotency genes *NANOG* and zinc finger protein 42 (*ZFP42*) and expression levels of neural lineage genes, such as paired box 6 (*PAX6*), microtubule associated protein 2 (*MAP2*), and neural cell adhesion molecule 1 (*NCAM1*) that were similar to or higher than those of AS iPSC-derived EBs.

**Figure 2:**
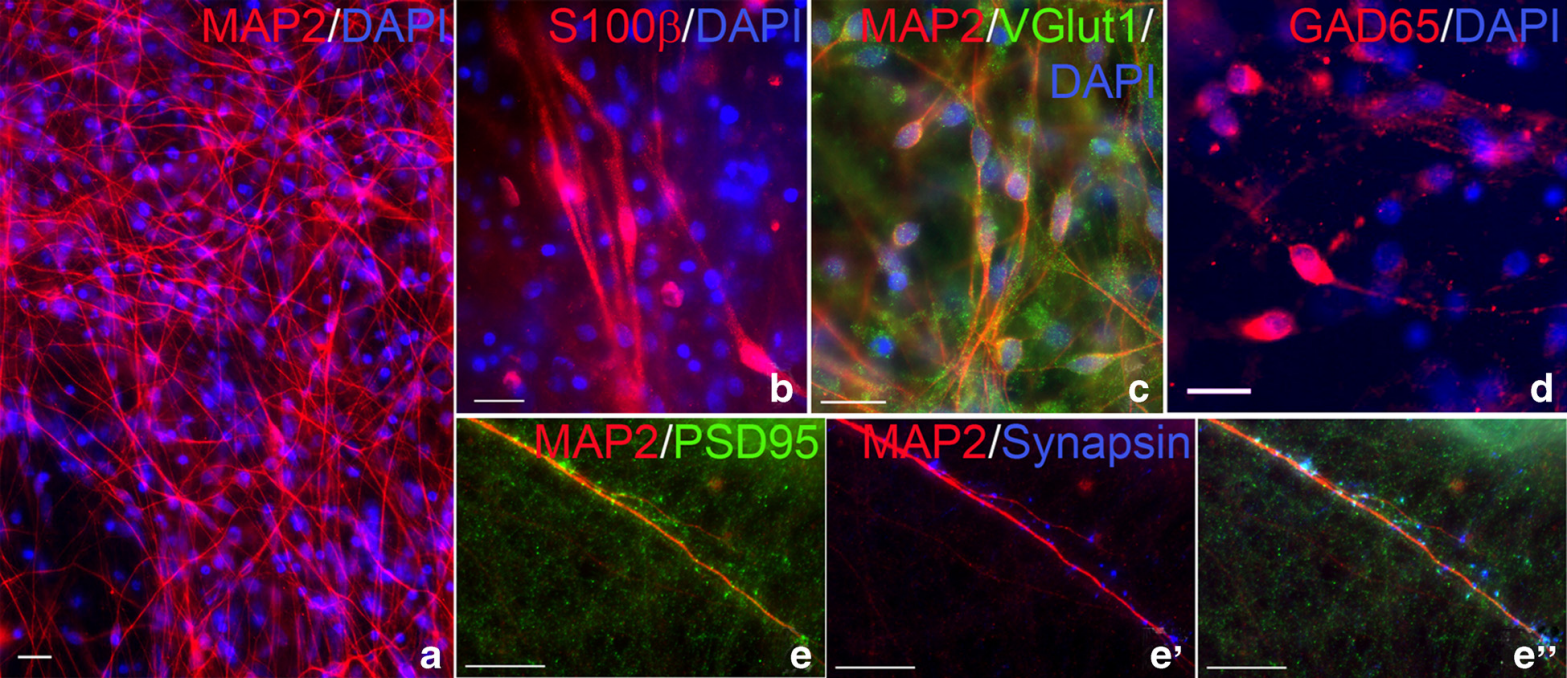
**Characterization of neurons derived from chromosome 15q11-q13.1 duplication syndrome (Dup15q) induced pluripotent stem cells (iPSCs). (a)** Immunocytochemistry for the neuronal marker MAP2 (red) indicates the presence of mature neurons in 10-week-old Idic1-8 iPSC-derived monolayer neuronal cultures. **(b)** S100β-positive astrocytes (red) are also present after 10 weeks of neural differentiation. **(c)** VGlut1-positive (green) excitatory and **(d)** GAD-65-positive (red) inhibitory neurons show a mixed population of neurons. **(e)** MAP2-positive (red) neurons are decorated with PSD-95 (green) and **(e’)** Synapsin (blue) puncta. **(e”)** Merged image showing co-localization of PSD-95 and Synapsin suggests the formation of functional synapses. Nuclei are counterstained with DAPI (blue). All scale bars are 20 μm.

The selected iPSC clones from each Dup15q genotype were then differentiated using either an EB-based differentiation protocol that mimics embryonic development or a modified monolayer neural differentiation protocol [19,20,31]. Both protocols generated neural progenitors and subsequently post-mitotic neurons from all genotypes. Analysis of 10-week-old monolayer neuronal cultures by immunocytochemistry demonstrated the cultures largely consisted of MAP2-positive neurons (Figure 2a). Astrocytes, labeled by S100 calcium binding protein beta (S100β), were also observed (Figure 2b). The neuronal population contained both vesicular glutamate transporter 1 (VGlut1)-positive excitatory neurons and glutamate decarboxylate 65 (GAD65)-positive inhibitory neurons (Figure 2c and Figure 2d). Co-localization of the postsynaptic density protein PSD-95 and the presynaptic marker Synapsin I along MAP2-positive neurites indicates the formation of functional synapses in 10-week-old neuronal cultures (Figure 2e-e”).

In order to compare neural cultures derived from the different cell lines used in this study, with respect to the types of cells present, we analyzed expression of neuronal (*βIII*-*tubulin*, RNA binding protein fox-1 homolog 3 (*RBFOX3*), and T-box brain 1 (*TBR1*)) and glial (*S100β*) genes in three independent neural cultures from each cell line. This analysis revealed that there was no significant difference in mean expression levels between cell lines [see Additional file 6: Figure S4A] (one-way ANOVA; *βIII*-*tubulin*: F_6,14_ = 0.9295, *P* = 0.5037; *RBFOX3*: F_6,14_ = 1.938, *P* = 0.1443; *TBR1*: F_6,14_ = 2.174, *P* = 0.1086; and *S100β*: F_6,14_ = 2.786, *P* = 0.0535). We also determined that all cell lines yielded neural cultures expressing similar levels of genes specific to excitatory (*VGLUT2*) and inhibitory (*GAD1*) neurons (Additional file 6: Figure S4A, one-way ANOVA; *VGLUT2*: F_6,14_ = 2.597, *P* = 0.0662 and *GAD1*: F_6,14_ = 2.932, *P* = 0.0456). Though the levels of expression varied between cell lines, we observed that all cell lines generated cells which expressed markers of dorsal forebrain including forkhead box G1 (*FOXG1*), *PAX6*, and orthdenticle homeobox 2 (*OTX2*) [see Additional file 6: Figure S4A] (one-way ANOVA; *FOXG1*: F_6,14_ = 11.75, *P* < 0.0001; *PAX6*: F_6,14_ = 3.878, *P* = 0.0172; and *OTX2*: F_6,14_ = 11.97, *P* < 0.0001). The midbrain specific gene engrailed 1 (*EN1*) was expressed in neural cultures from all cell lines but at noticeably lower levels than the forebrain-specific genes [See Additional file 6: Figure S4A] (one-way ANOVA, F_6,14_ = 7.664, *P* = 0.0009). We also assayed for expression of NK2 homeobox 2 (*NKX2.1*), a marker of ventral forebrain progenitors, and found that its levels were very low or undetectable in most cultures. Importantly, the replicate neural cultures used in these experiments included those derived by both monolayer culture and EB differentiation, demonstrating that there were no significant differences in the types of cells generated between the two protocols.

### Electrophysiological recordings of idic(15) iPSC-derived neurons

**Figure 3:**
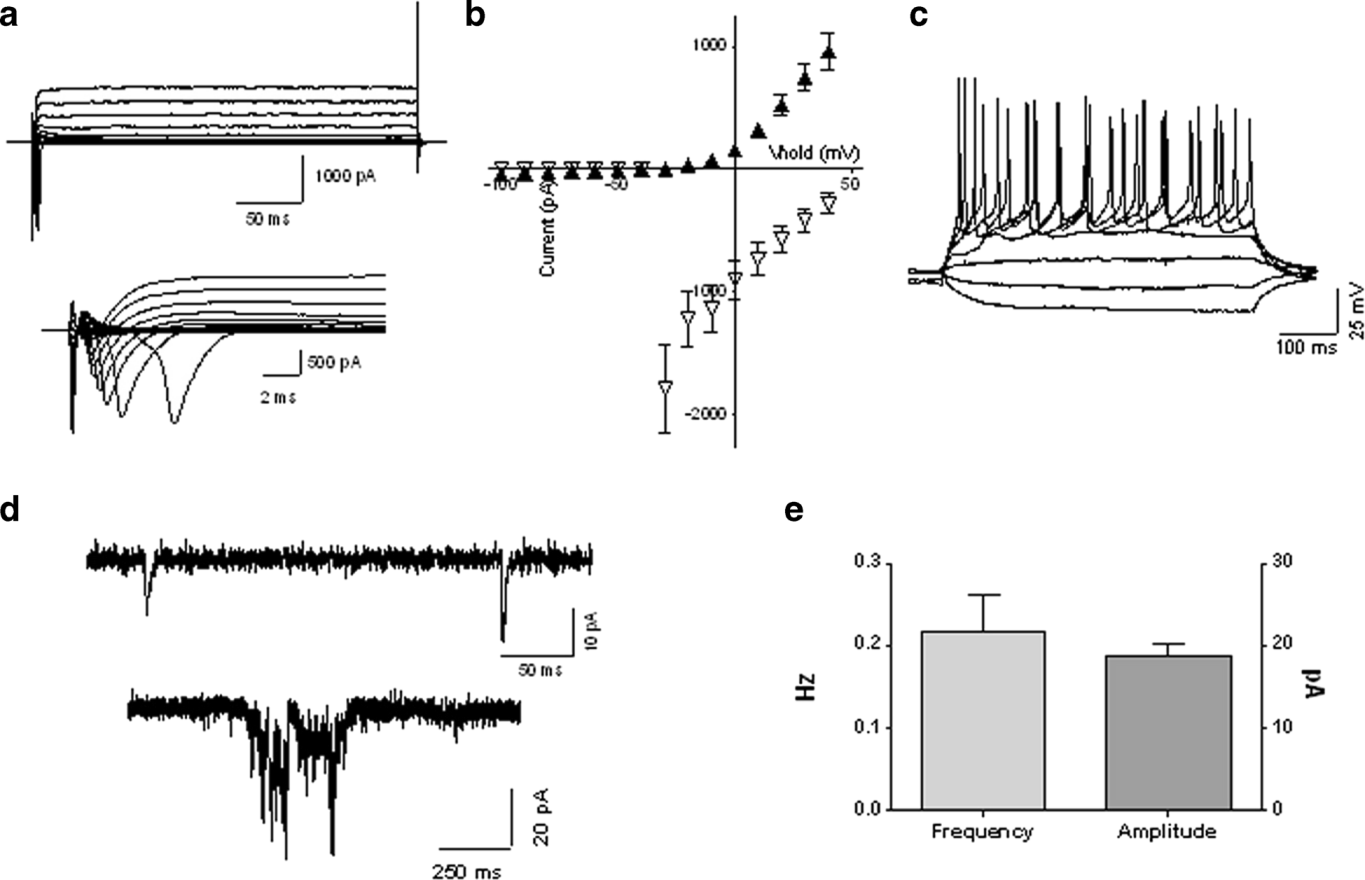
**Electrophysiological characterization of induced pluripotent stem cell (iPSC)***-***derived neurons from a chromosome 15q11-q13.1 duplication syndrome (Dup15q) subject. (a)** Representative whole cell potassium currents (top) and sodium currents (bottom) recorded from a 12-week-old Idic1-8 iPSC-derived neuron generated using the embryoid body based differentiation protocol. **(b)** Current-voltage relationship for peak sustained K current (filled triangles) and peak Na current (open triangles) from 12-week-old neurons (n = 16). **(c)** Representative action potential train from a 12-week-old neuron. **(d)** Example sweeps of excitatory synaptic currents recorded from a 14-week-old neuron. **(e)** Group data showing average frequency and amplitude of synaptic currents in 14 week-old-neurons (n = 20).

We carried out electrophysiological recordings from differentiated neurons derived from the idic(15) iPSC line (Idic1-8) during *in vitro* development using the EB differentiation protocol. In 3-week-old cultures, most neurons (21/24 cells) fired a single mature action potential in response to depolarizing current injection. In 6-week-old cultures, trains of action potentials were induced in more than half of the neurons recorded (29/51 cells). At weeks 12 to 14, a similar percentage of cells fired trains of mature action potentials and displayed large (>1 nA) inward and outward currents in response to depolarization (Figure 3a-c). Inward sodium currents and action potentials were blocked by the sodium channel blocker tetrodotoxin (1 μM; data not shown). Spontaneous excitatory synaptic events were seen in about half of the neurons in 6-week-old cultures (28/52 cells), with an average frequency of 0.11 ± 0.03 Hz (n = 28). In 14-week-old cultures, as shown in Figure 3d-e, isolated synaptic currents and occasional bursts of activity were seen in the majority of cells at a slightly higher frequency (0.22 ± 0.05 Hz; n = 20). Spontaneous excitatory synaptic currents were blocked by the AMPA receptor antagonist 6-cyano-7-nitroquinoxaline-2,3-dione (CNQX) (10 μM; data not shown). These data indicate that Dup15q neurons show *in vitro* maturation of intrinsic excitability and evidence of functional synapses, laying the groundwork for a detailed comparison of functional properties among different genotypes.

### Expression of 15q11-q13.1 genes in Dup15q iPSCs and iPSC-derived neurons

The 15q11-q13.1 region includes several genes involved in neuronal structure and function (Figure 1a). Imprinting in the 15q11-q13.1 region results in allele-specific expression of several genes such that in non-neuronal cells (including pluripotent cells), *SNRPN* is expressed from the paternal allele and *UBE3A* is expressed from both parental alleles. In neurons, however, *SNRPN* is still expressed solely from the paternal allele, but *UBE3A* becomes paternally silenced and is expressed only from the maternal allele [32]. While some studies suggest that *ATP10A* is maternally expressed in the brain, others suggest that it is biallelically expressed or that its imprinting status varies among individuals [33]. Other genes in the region, including *TUBGCP5*, *CYFIP1*, *NIPA1*, *GABRB3*, *HERC2*, and *CHRNA7* are expressed biallelically in all cell types tested to date. Evidence from analysis of both Dup15q lymphoblast and postmortem brain tissue samples suggests that expression levels of these genes differ significantly from normal individuals [34,35]. To investigate whether copy number variations of the 15q11-q13.1 region result in corresponding changes in the expression of genes located in the region, we performed qRT-PCR with iPSCs and iPSC-derived neurons (both monolayer and EB-derived) from all genotypes for *TUBGCP5*, *CYFIP1*, *NIPA1*, *SNRPN*, *UBE3A*, *ATP10A*, *GABRB3*, *HERC2*, *and CHRNA7* [see Additional file 7: Figure S5A-B]. In each case, we compared the expression level of the gene in the disease-specific cells to the normal cells.

**Figure 4:**
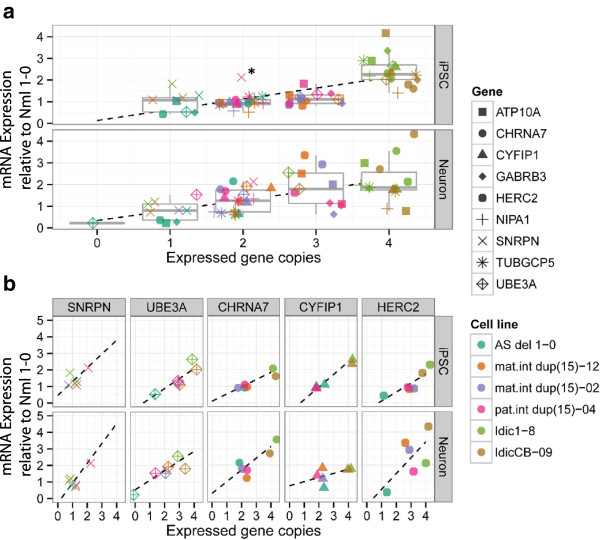
**Expression of 15q11-q13.1 genes in chromosome 15q11-q13.1 duplication syndrome (Dup15q) induced pluripotent stem cells (iPSCs) and iPSC-derived neurons. (a)** Box plots depicting the distribution of average mRNA expression levels of 15q11-q13.1 genes in Dup15q iPSCs (top) and 10-week-old iPSC-derived neurons (bottom) relative to normal samples (Nml 1-0) as they correspond to the number of expressed copies of each gene. The dark gray line in the center of each box represents the median of the average relative mRNA expression levels of genes containing each number of expressed gene copies. Boxes indicate the 25th, 50th, and 75th percentiles. Whiskers represent the 5th and 95th percentiles. The data point for *SNRPN* in the pat. int dup(15)-04 iPSCs (marked by asterisk) is not indicative of unexpected mRNA expression levels relative to gene copy number, since this paternally expressed gene is duplicated on the paternal allele in this sample. **(b)** The average mRNA expression levels for selected 15q11-q13.1 genes in Dup15q iPSCs (top) and 10-week-old iPSC-derived neurons (bottom) relative to that of normal (Nml 1-0) iPSCs and neurons is presented in relation to the respective number of expressed copies of each gene.

For all genotypes, expression of the 15q11-q13.1 genes in iPSCs generally corresponded to expressed copy number when compared to iPSCs derived from a control individual (Nml 1-0) (Figure 4a, top panel). The levels of expression of paternal-specific *SNRPN* in AS iPSCs and in the maternal int dup (15) and idic(15) iPSCs were similar to normal iPSCs because they only have a single active copy of the gene. The paternal int dup(15) iPSCs showed a twofold increase in *SNRPN* expression compared to normal iPSCs, reflecting the two active copies of the gene (Figure 4a, top panel; Additional file 7: Figure S5A). *UBE3A*, which is not imprinted in iPSCs was expressed at approximately half the level of normal in AS iPSCs and between two- and threefold of normal in idic(15) iPSCs. While *UBE3A* expression fell within the range of normal expression in both iPSC lines derived from the maternal int dup(15) sample and the paternal int dup(15) iPSCs, the average *UBE3A* expression level in all three of these iPSC lines was consistently higher than that of normal iPSCs.

The expression levels of *HERC2* and *NIPA1* were not as predicted from copy number in some of the iPSC lines. *HERC2* was expressed at levels near or slightly less than those seen in normal iPSCs in both the paternal and maternal int dup(15) iPSCs (Figure 4b, top panel; Additional file 7: Figure S5A). Expression of *NIPA1* was roughly half that of normal iPSCs in both clones of the maternal int dup(15) iPSC lines [see Additional file 7: Figure S5A].

Expression levels of most 15q11-q13.1 genes in 10-week-old AS and idic(15) iPSC-derived neurons also largely correlated with their respective expressed copy numbers (Figure 4a, bottom panel), with one notable exception. *HERC2* expression in one of the idic(15) cell lines (IdicCB-09) was nearly 4.5-fold higher than normal, which is a deviation from the expected twofold increase due to these cells having two additional copies of the gene (Figure 4b, bottom panel, Additional file 7: Figure S5B). *UBE3A* is imprinted in iPSC-derived neurons and, accordingly, *UBE3A* expression levels were less than 25% of normal levels in AS iPSC-derived neurons. In idic(15) iPSC-derived neurons, *UBE3A* levels were about 2.5-fold higher than normal neurons, as expected for cells with three active copies of the gene (Figure 4b, bottom panel; Additional file 7: Figure S5B).

In the maternal int dup(15) iPSC-derived neurons (mat. int dup(15)-02), we found that *UBE3A* was expressed at the predicted 1.5-fold over normal. However, *UBE3A* was also increased to a similar degree in the isogenic normal (mat. int dup(15)-12) neurons. In order to understand this apparent discrepancy in expected *UBE3A* expression, we analyzed *UBE3A* copy number in the maternal int dup(15) and isogenic normal clones using a qPCR CNV assay, and determined that both cell lines had three copies of *UBE3A* [see Additional file 8: Figure S6A]. In addition, we detected RNA expression from three *UBE3A* alleles by RNA-FISH with a *UBE3A* BAC probe in iPSCs of both cell lines [see Additional file 8: Figure S6B]. This suggests that while initial analysis of iPSC clones, using DNA FISH for *SNRPN* and the PWS-IC methylation assay, indicated that the mat. int dup(15)-12 clone did not contain the 15q11-q13.1 duplication, this clone was also mosaic and over approximately 25 passages resolved to consist mostly of duplication-carrying cells. Therefore, we can interpret data generated using this cell line as a second maternal interstitial duplication clone.

15q11-q13.1 gene expression in the maternal int dup(15) and paternal int dup(15) iPSC-derived neurons did not correlate well with expressed copy number for several genes (Figure 4a, bottom panel). *HERC2* levels were threefold higher than normal in maternal int dup(15) neurons, as opposed to the predicted 1.5-fold increase from the three expressed copies of the gene in these cells (Figure 4b, bottom panel). We also observed unexpected increases in expression of *CYFIP1* and *CHRNA7* in both the maternal and paternal int dup(15) iPSC-derived neurons (Figure 4b, bottom panel). We did observe the expected increase in *SNRPN* expression in the paternal int dup(15) neurons, however, expression of *NIPA1*, *UBE3A*, *HERC2*, and *CHRNA7* were also higher than expected based on expressed copy number.Together, these data suggest that gene expression level correlates well with expressed copy number in iPSCs but less so in iPSC-derived neurons (compare top and bottom panels of Figure 4a). Furthermore, interstitial duplication iPSCs and neurons are more likely to have gene expression levels different from expected levels based on expressed copy number.

### Transcriptome analysis of AS, normal, and idic(15) iPSC-derived neurons

We also sought to analyze gene expression genome-wide. To test the hypothesis that alterations in gene expression would reflect either 1) disrupted pathways caused by altered dosage of the duplicated 15q region or 2) the transcriptional responses to the physiological changes in the AS and idic(15) neurons, we performed strand-specific mRNA-Seq on 10-week old neurons generated from AS, normal, and idic(15) (Idic1-8) iPSCs. mRNA-Seq data were generated using neurons derived from two independent rounds of *in vitro* differentiation using the EB protocol from iPSCs of each of the three genotypes. Gene expression levels were compared between biological replicates and were found to be highly similar [see Additional file 9: Table S3, r^2^ ≥ 0.97]. The gene expression levels for the replicates were averaged and the AS and idic(15) samples were each compared to the normal sample.

**Figure 5:**
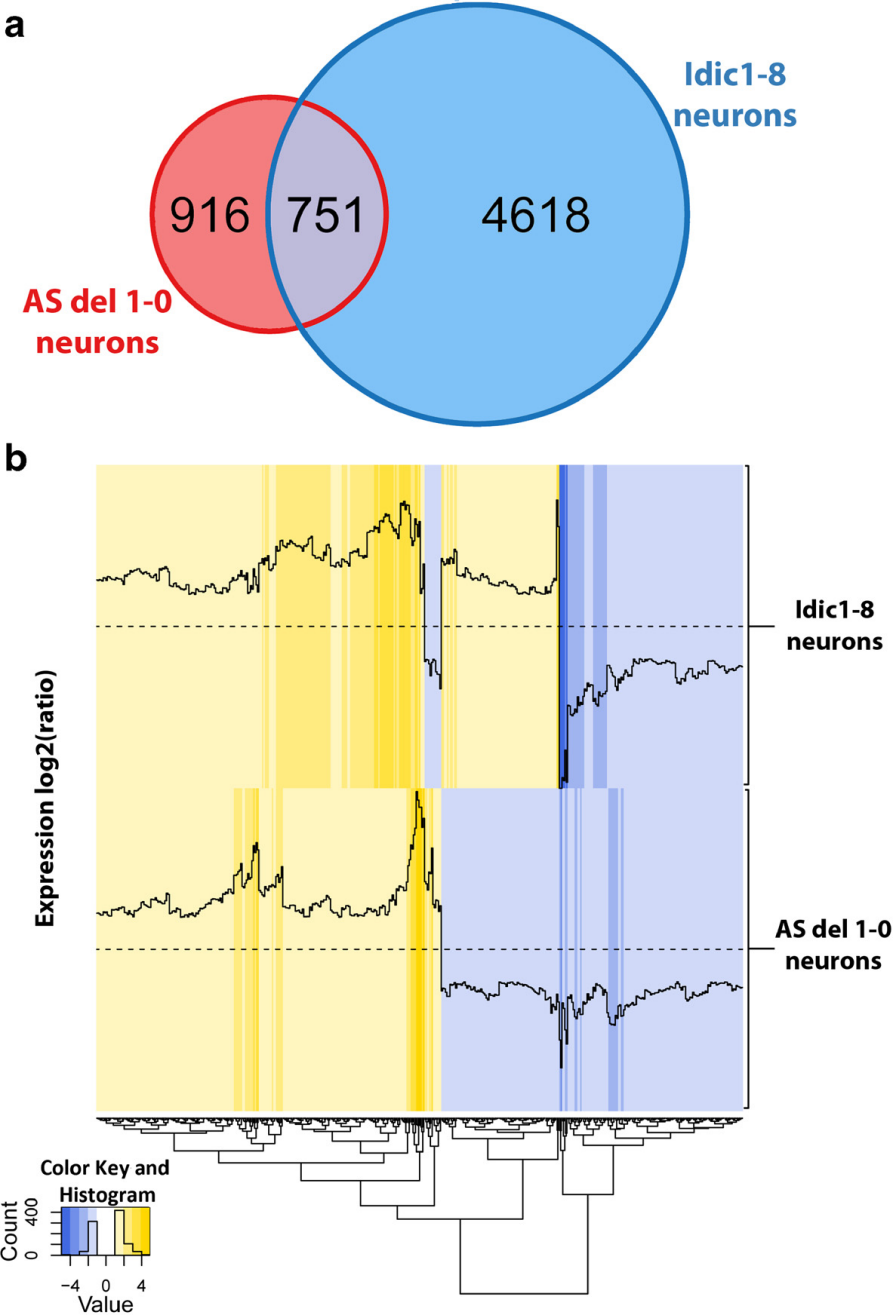
**Most of the genes differentially expressed in both Angelman syndrome (AS) and isodicentric chromosome 15 (idic(15)) induced pluripotent stem cell (iPSC)-derived neurons compared to normal are changed in the same direction. (a)** Venn diagram depicting the number of genes differentially expressed (twofold up or down) between AS (AS del 1-0) and idic(15) (Idic1-8) iPSC-derived neurons compared to normal (Nml 1-0) iPSC-derived neurons. The overlap of the two circles represents the set of shared differentially expressed genes in both AS and idic(15). **(b)** Heat map showing the mRNA expression levels relative to normal (Nml 1-0) of the top 200 genes differentially expressed in both AS (AS del 1-0) and idic(15)(Idic1-8) iPSC-derived neurons. Genes are clustered according to their similarity in gene expression levels across both samples. Genes colored in yellow show expression levels greater than normal, and genes colored in blue show expression levels less than normal. The trace lines indicate the expression levels of individual genes relative to normal. Normal expression level is indicated by the straight dotted lines.

Genes showing a twofold increase or decrease in expression compared to the normal sample were considered differentially expressed between those two samples. 5,369 genes were differentially expressed between idic(15) and normal iPSC-derived neurons (Figure 5a). A total of 1,667 genes were found to be differentially expressed between AS and normal iPSC-derived neurons (Figure 5a). Of these, 751 genes were differentially expressed in both samples compared to normal (Figure 5a). The number of genes found to be differentially expressed in both samples is greater than expected by chance (hypergeometric distribution with population size = 27,297; *P* = 1.88 X 10^-131^). Moreover, 76% of the genes differentially expressed in both AS and idic15 neurons show the same tendency (that is, both upregulated or both downregulated). Figure 5b shows a heat map of the top 200 most differentially expressed genes in both samples. Gene Ontology analysis was performed on the lists of genes specifically up- or downregulated in AS and idic(15) neurons compared to normal neurons [see Additional file 10: Table S4, Additional file 11: Table S5, Additional file 12: Table S6, and Additional file 13: Table S7]. The gene ontology term ‘neuronal differentiation’ was the most significantly enriched category from the lists of genes downregulated in both AS and idic(15) neurons (*P* = 9.7 X 10^-10^ and *P* = 4.31 X 10^-8^ for AS and idic(15) neurons, respectively). Genes involved in cell cycle (*P* = 1.64 X 10^-23^) and protein catabolic processes (*P* = 5.96 X 10^-19^) were enriched among the list of genes upregulated in idic(15) neurons.

**Figure 6:**
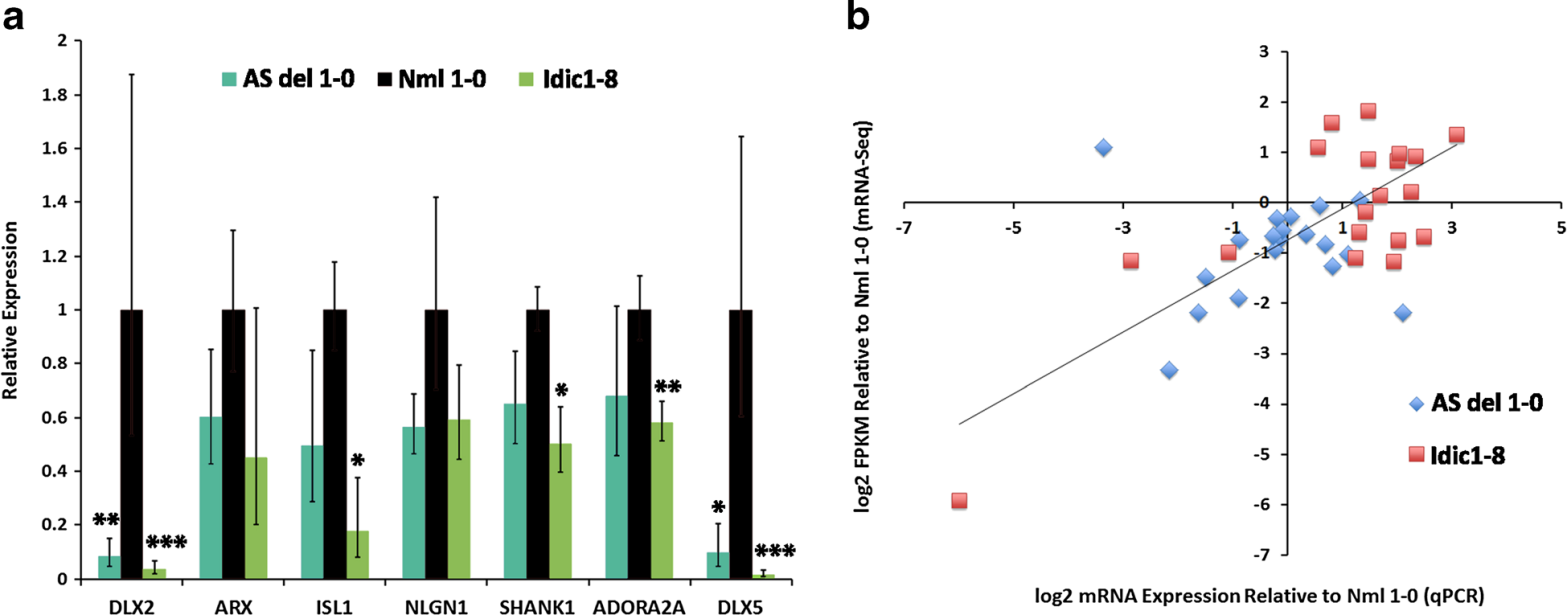
**Quantitative reverse transcription PCR (qRT-PCR) validation of mRNA-Seq data. (a)** Differential gene expression of select autism candidate genes and genes implicated in seizure disorders were verified by qRT-PCR in 10-week-old AS (AS del 1-0), normal (Nml 1-0), and idic(15) (Idic1-8) iPSC-derived neurons. *P* values: *P ≤0.1, **P ≤0.05, ***P ≤0.01. **(b)** Correlation of relative expression levels for 19 genes in iPSC-derived neurons as determined by mRNA-Seq and qRT-PCR. The log2-transformed RQ values for mRNA expression relative to normal neurons are plotted on the x-axis against the log2-transformed FPKM values obtained by mRNA-Seq analysis (y-axis).

Interestingly, several known autism and epilepsy candidate genes, namely distal-less homebox 2 (*DLX2*), aristaless related homeobox (*ARX*), ISL LIM homeobox 1 (*ISL1*), neuroligin 1 (*NLGN1*), SH3 and multiple ankyrin repeat domains 1 (*SHANK1*), adenosine A2A receptor (*ADORA2A*), and distal-less homeobox 5 (*DLX5*), were found in the list of genes differentially expressed and downregulated in AS or idic(15). To validate the transcriptome data and further interrogate some of these genes, qRT-PCR was performed on RNAs isolated from normal, AS and idic(15) (Idic1-8) neurons (Figure 6a). All seven of the genes assayed were shown to be downregulated in both AS and idic(15) iPSC-derived neurons. qRT-PCR data from 19 different genes demonstrated agreement between qRT-PCR and mRNA-Seq data (Figure 6b). Although maternal int dup(15) iPSC-derived neurons were not included in the whole transcriptome analysis, we also assayed expression of the selected autism and epilepsy candidate genes in these neurons by qRT-PCR. We observed that, with the exception of *ADORA2A*, expression of these genes was also downregulated in maternal int dup(15) iPSC-derived neurons when compared to normal neurons [see Additional file 14: Figure S7].

### Rescue of normal *UBE3A* expression levels in idic(15) iPSC-derived neurons

**Figure 7:**
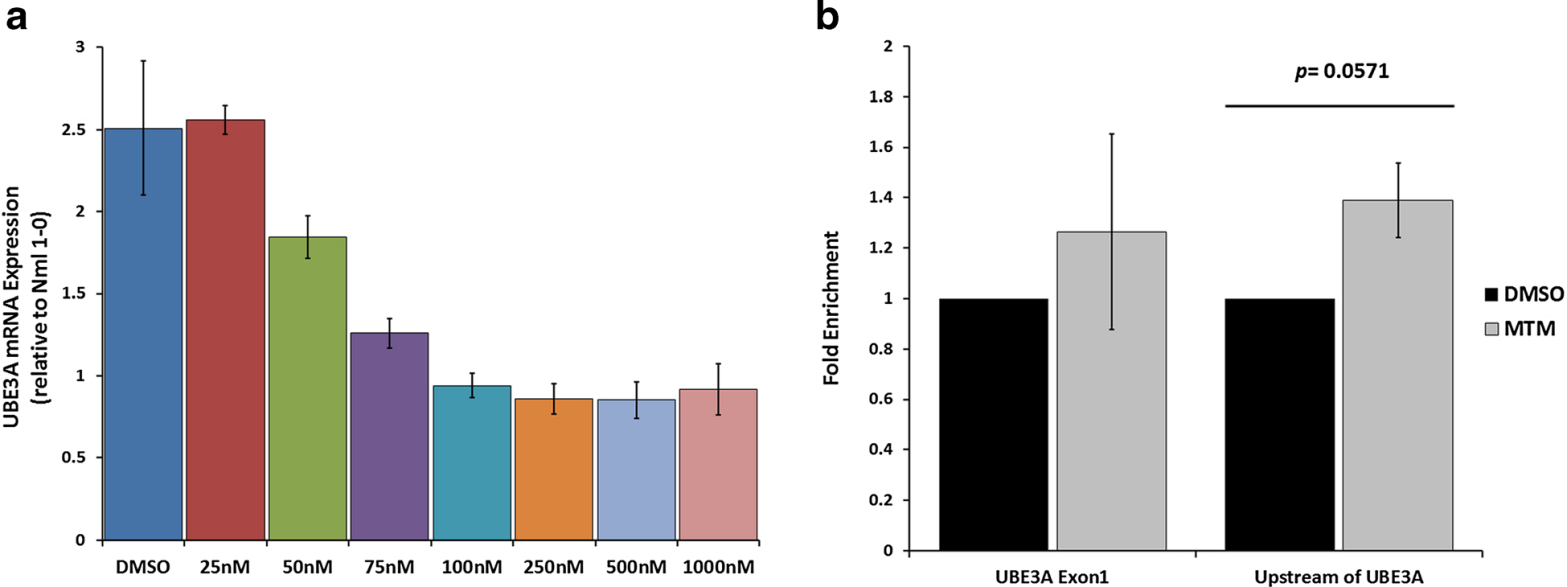
**Rescue of normal *UBE3A *expression levels following treatment of isodicentric chromosome 15 (idic(15)) induced pluripotent stem cell (iPSC)-derived neurons with mithramycin. (a)** qRT-PCR analysis of *UBE3A* mRNA expression in 10-week-old idic(15) (Idic1-8) iPSC-derived neurons following 72 hours of treatment with DMSO (vehicle) or mithramycin at concentrations ranging from 25 nM to 1 μM. Data are presented as mean expression levels relative to that of untreated normal (Nml 1-0) iPSC-derived neurons. **(b)** ChIP-qPCR analysis of YY-1 binding at two sites in UBE3A, one within exon 1 and one upstream of exon 1, in 10-week-old idic(15) (Idic1-8) iPSC-derived neurons following 72 hours of treatment with either DMSO or 100 nM mithramycin.

To investigate whether the aberrant expression levels of *UBE3A* in idic(15) neurons could be pharmacologically manipulated, we treated 10-week-old idic(15) iPSC-derived monolayer neuron cultures with the DNA-binding compound mithramycin. This drug binds to GC-rich sequences in specific gene promoters and competes with transcription factors that regulate gene expression [36,37]. We reasoned that since the *UBE3A* promoter is GC-rich and is known to be occupied by many transcription factors in various cell types [38], competition for binding by mithramycin may alter the levels of *UBE3A* transcription. Following 72 hours of drug treatment, we found that *UBE3A* mRNA levels in idic(15) neurons were reduced in a dose-dependent manner to levels near that of normal iPSC-derived neurons (Figure 7a). These data suggest that *UBE3A* can be pharmacologically regulated at the transcriptional level by mithramycin. However, competition for transcription factor binding by mithramycin is not specific for *UBE3A* and occurs at many gene promoters. In line with this, we detected changes in expression levels of several other genes following mithramycin treatment, including the other 15q11-q13.1 genes *SNRPN*, *GABRB3*, and *CYFIP1* [see Additional file 15: Figure S8].

In an effort to identify possible mechanisms whereby mithramycin treatment rescues *UBE3A* expression levels, we assayed transcription factor binding to *UBE3A* by ChIP. It is well established that mithramycin inhibits binding of the Sp1 transcription factor and affects transcription of Sp1 target genes [37,39]. We were unable to detect Sp1 enrichment at several sites analyzed within the *UBE3A* promoter [see Additional file 16: Figure S9A]. For this reason, we instead analyzed binding of the transcription factor yin yang 1 (YY-1), which we confirmed does bind within the *UBE3A* promoter in both iPSCs and iPSC-derived neurons [see Additional file 16: Figure S9B]. YY-1 can act both as a transcriptional activator and repressor [40]. Transcriptional repression by YY-1 is thought to be mediated by recruitment of histone deacetylases and histone acetyltransferases [41]. We assayed binding of YY-1 to *UBE3A* at two sites, one within exon 1 and one about 250 base pairs upstream of exon 1, following treatment of idic(15) iPSC-derived neurons for 72 hours with either 100 nM mithramycin or DMSO. While there was no significant difference between the treatments in binding of YY-1 at exon 1, binding at the upstream site was increased in mithramycin-treated neurons to roughly 1.4-fold that of DMSO-treated neurons (Figure 7b, *P* = 0.0571). These data suggest that mithramycin treatment may recruit YY-1 to the *UBE3A* promoter, where it can act in a repressive manner to decrease *UBE3A* expression levels.

## Discussion

Dup15q syndrome is a genetic form of autism in which the features of affected individuals closely resemble those in idiopathic autism [1,2,8,9]. In addition to the impairments in social interaction, verbal and nonverbal communication, and stereotyped behavior that underlie autism, individuals with Dup15q syndrome also suffer from hypotonia, seizures, developmental delay, and behavioral problems. We have generated iPSC models of Dup15q syndrome from a variety of patient samples containing different CNVs of the 15q11-q13.1 region. We show that the imprinting status of the PWS-IC is maintained following reprogramming of patient samples and that the ratio of methylated to unmethylated DNA at the PWS-IC confirms the expected 15q11-q13.1 copy number in each patient-derived iPSC line. Importantly, we demonstrated that all of the iPSC lines we generated can be differentiated into functionally mature neurons, as indicated by immunocytochemistry and electrophysiology. The ability to generate functional human neurons in large numbers from these iPSCs will enable us to perform molecular and cellular phenotypic analyses, and to test candidate therapeutic compounds in live human Dup15q neurons. Thus, these cell lines are an important resource for understanding Dup15q syndrome as well as idiopathic autism.

In contrast to AS, a single gene disorder which can result from loss of function of the *UBE3A* gene alone, it is not known whether Dup15q syndrome results from overexpression of *UBE3A* or from overexpression of *UBE3A* plus one or more other genes in the 15q11-q13.1 region. Since maternal, but not paternal duplications typically lead to the Dup15q syndrome, a role for *UBE3A* seems highly likely. However, the contribution of other genes has been difficult to explore since the only patient specimens available were blood or post-mortem tissues that may not accurately reflect the gene expression observed in live neurons. Our iPSC models of Dup15q allow for gene expression analysis in live human neurons carrying the various 15q11-q13.1 CNVs. This experimental system also permits us to better resolve some of the confounding issues involved with post-mortem brain samples (variable mRNA or protein quality) and with genetically engineered transformed cell lines (unknown genetic and epigenetic aberrations) that have been previously used to study Dup15q syndrome [34,42].

Our analysis of 15q11-q13.1 gene expression in undifferentiated iPSCs showed that, on the whole, expression levels in all genotypes correlated with 15q11-q13.1 copy number, including proper allele-specific expression of imprinted *SNRPN*. Consistent with previous findings in patient lymphoblasts [35], fibroblasts [43], and post-mortem brain [34], we confirmed increased expression of *UBE3A* in iPSCs derived from two separate idic(15) individuals. We also observed increased expression of several other genes included in the duplication region such as *CYFIP1*, *GABRB3*, and *HERC2*.

We found that following differentiation of iPSCs into functionally mature neurons, 15q11-q13.1 transcript levels did not consistently correlate with copy number. For example, *CYFIP1* expression was increased relative to normal in both maternal and paternal int dup(15) neurons, which do not have a duplication of *CYFIP1*. Meanwhile, *TUBGCP5* levels were significantly reduced in maternal int dup(15) neurons. In addition, a striking threefold increase in non-imprinted *HERC2* expression was observed in maternal int dup(15) neurons, while the predicted expression level was 1.5-fold of normal. Interestingly, we also observed slight increases in expression of *CHRNA7*, a gene located well outside of the duplication region in both maternal and paternal int dup(15) neurons. AS iPSC-derived neurons, in which *CHRNA7* copy number is unaffected, also showed a significant twofold increase of *CHRNA7* compared to normal neurons. Together, these data suggest that, in neurons, transcription of genes both within and adjacent to the 15q11-q13.1 duplication region is not correlated to gene copy number. There are at least three possible reasons for these unexpected gene expression changes: 1) individual-to-individual variability; 2) disruption of long-distance regulatory elements by the chromosomal duplications; or 3) aberrant function of the neurons not associated with the chromosomal rearrangement.

Normal individual-to-individual variability is a confounding problem for gene expression studies in any human tissue type or patient-derived cell line. In some cases, the variability between individuals can be significant due to the different genetic backgrounds. We cannot rule out the possibility that the unexpected gene expression changes that we observe are due to normal variation. This appears unlikely, however, since the gene expression in the Dup15q iPSCs very closely reflects the copy number of the interrogated genes. The problems associated with normal variation can be addressed by examining samples from multiple individuals or by comparing isogenic Dup15q and normal cell lines. We have compared gene expression in iPSC-derived neurons from two different idic(15) individuals, and they show similar expression levels of all genes, except for *ATP10A* and *HERC2. ATP10A* is highly variable between replicate neurons from the same individual and has proven difficult to analyze even in mouse models where the genetic background is the same [44]; thus, changes in its expression levels are difficult to interpret. *HERC2* encodes a ubiquitin ligase that has been shown to act in a complex with UBE3A [45]. The increased *HERC2* levels could conceivably influence the severity of the symptoms in some idic(15) individuals. Isogenic cell lines can be obtained by deriving Dup15q and normal cell lines from the same mosaic individual, as attempted for our maternal interstitial duplication individual, or by genetically correcting the duplication in individual cell lines. These comparisons will be important future studies for Dup15q syndrome.

Our results indicate that the correlation between gene expression level and copy number is different in interstitial versus isodicentric 15q11-13.1 duplications, and we suspect that alterations in long range chromatin regulation may be involved. Several models have been proposed to explain how deletions and duplications of genes or large regions of DNA can alter transcription of a given gene. In particular, regulatory sequences can be disrupted by direct mutation of promoters and enhancers. Alternately, binding sites for proteins that mediate the necessary chromatin looping required for proper gene expression can be disrupted. Abrogation of long-distance transcriptional regulation due to chromosomal rearrangements has been implicated in several human diseases [46], and our data may suggest such a mechanism in Dup15q syndrome. We hypothesize that chromatin-organizing regulatory elements exist near the duplication/deletion breakpoints. The function of these regulatory elements may be disrupted by the duplication or deletion events. The effect of this regulatory disruption is predicted to affect interstitial duplications and deletions more than idic(15) samples, consistent with our results.

It is possible, however, in neurons that undergo silencing of the paternal *UBE3A* allele and complex changes in chromatin structure that transcription from the extra maternal *UBE3A* alleles is subject to differential regulation [47]. Studies using an engineered neuroblastoma cell line model of Dup15q showed aberrant expression levels of several 15q11-q13.1 genes compared to levels predicted by copy number [42]. Meguro-Horike *et al*. propose that the discordance in transcript levels compared to copy number of 15q11-q13.1 genes is a result of a disruption of transcriptional regulation normally mediated either by pairing of homologous chromosomes or by intra-chromosomal pairing in cells containing extra 15q11-q13.1 alleles [42].

We also investigated genome-wide gene expression differences between idic(15), AS, and normal iPSC-derived neurons. Altered *UBE3A* gene dosage is proposed to underlie most of the phenotypic consequences of Dup15q syndrome [4,8,14]. We reasoned that gene expression changes that are caused by increases in *UBE3A* expression in Dup15q neurons should be reversed in AS neurons that have decreased *UBE3A* expression. In addition to its role in ubiquitination, *UBE3A* acts as a transcriptional co-activator. UBE3A can also affect gene transcription via ubiquitination of protein targets in signaling pathways or by directly changing expression levels of individual genes. In both cases, the *UBE3A*-mediated effects on transcription should be opposite between idic(15) and AS neurons. As expected, there were more changes between idic(15) and normal than AS and normal neurons. This is likely due to the fact that the idic(15) neurons have two extra copies of a 9.6 Mb region, while AS neurons have one less copy of a 5.1 Mb region.

When considering the genes differentially expressed in both AS and idic(15) neurons compared to normal neurons, we found more similarities than differences. A remarkable 75% of all genes found differentially expressed and common to both idic(15) and AS neuron samples showed a similar expression pattern (that is, both downregulated or both upregulated), contrary to our hypothesis that AS and idic(15) are ‘opposite’ disorders. In fact, the same gene ontology term was identified amongst genes downregulated in both AS and idic(15) neurons. Since these gene expression changes represent changes at the mRNA level and not necessarily the protein level, we speculate that the shared changes result from a common neuronal response to malfunctioning synapses. We suspect that these gene expression changes are secondary to the chromosomal duplication or deletion, and may even occur in neurodevelopmental disorders not involving the chromosome 15q11-q13.1 region. Consistent with this idea, the M12 gene module identified by Voineagu *et al*. [48], that is transcriptionally downregulated in postmortem brain samples from idiopathic autism cases shares similar gene ontology terms to those downregulated in idic(15) and AS neurons, including terms involving synapses, axons, and neuron projections.

The major categories of genes upregulated in idic(15) neurons include genes involved in the cell cycle and those involved in protein catabolism, including those involved in ubiquitin-mediated proteolysis. Why the cell cycle would be affected in postmitotic neurons is perplexing. Growing evidence suggests that abnormal expression of cell cycle genes can participate in neuronal death, but neither increased nor decreased neuronal death has been observed in Dup15q syndrome or in our cell culture models. A recent report showed that the retinoblastoma 1 (RB1) protein is required for continuous cell cycle repression and neuronal survival [49], and it is becoming more apparent that post-mitotic neurons actively repress the cell cycle [50]. It is possible that the upregulated cell cycle genes serve to repress the cell cycle and may represent more rapid maturation of the idic(15) *in vitro* differentiated neurons. Indeed, *RB1* is one of the upregulated cell cycle genes. There are also several proteasome subunit and ubiquitin-mediated proteolysis genes that are included among the cell cycle genes, thereby contributing to the significance of this gene ontology term. The upregulation of genes involved in protein catabolism and ubiquitin mediated proteolysis is somewhat expected, although it is interesting that a large number of genes involved in this pathway are upregulated. Ube3a was recently shown to regulate protein homeostasis by directly ubiquitinating Rpn10, a proteasomal shuttling protein, in *Drosophila melanogaster*[51]. If Rpn10 is shown to be a target of UBE3A in human neurons, this could explain the dysregulation of protein catabolism in idic(15) neurons.

We sought to reduce *UBE3A* mRNA levels in idic(15) neurons. Using our iPSC model of idic(15), we were able to perform a proof-of-principle experiment to determine whether *UBE3A* levels could be pharmacologically reduced in human neurons. We found that treatment of idic(15) iPSC-derived neurons with the DNA binding compound mithramycin, an anti-tumor antibiotic, was able to reduce *UBE3A* mRNA to levels similar to that of normal neurons. While several studies have shown that mithramycin inhibits transcription by competing for binding at GC-rich regions with the transcription factor Sp1 [37,39], we were unable to demonstrate Sp1 binding at *UBE3A* by ChIP in our neurons after trying three different anti-Sp1 antibodies. We did, however, make the novel observation that binding of YY-1 is increased following mithramycin treatment. Since YY-1 can act as both a transcriptional activator and repressor [40], we hypothesize that YY-1 acts in a repressive manner at *UBE3A* as increased binding is associated with a reduction in *UBE3A* transcript following mithramycin treatment. YY-1 mediated transcriptional repression has been shown to involve recruitment of histone deacetylases and histone acetyltransferases and thereby establishment of repressive chromatin modifications [41]. While mithramycin is an FDA-approved drug used in the treatment of various cancers [52-54], we do not suggest that it is suitable for use in the clinical treatment of Dup15q, due to its effects on the expression of many other genes. Our findings do demonstrate the feasibility of using Dup15q iPSC-derived neurons to screen for other therapeutic compounds with the aim of restoring normal *UBE3A* expression levels.

## Conclusions

We have derived iPSC lines from two idic(15) individuals, one maternal int dup(15) individual and one paternal int dup(15) individual. These iPSCs can be differentiated into electrophysiologically mature neurons. These cell lines are an important resource for studies of Dup15q syndrome and idiopathic autism. Our analysis of gene expression in iPSCs and iPSC-derived neuronal cultures containing varied CNVs of the 15q11-q13.1 region supports the theory that altered expression of several genes in this region may underlie the Dup15q phenotype. While altered *UBE3A* gene dosage is often proposed to be the mechanism responsible for Dup15q syndrome, our data along with data from other studies [8,34,42] suggest that disruption of additional candidate genes may contribute significantly. Altered expression of such additional genes may be due to either their presence in the duplication region or to disruption of their transcriptional regulation as a result of the chromosomal rearrangement (and thus independent of copy number). As a result, our data also point to possible disruption of transcriptional regulation of genes in the duplication region in iPSC-derived neurons. Finally, we restored *UBE3A* mRNA levels in idic(15) neurons to normal levels using the antitumor antibiotic, mithramycin, demonstrating that *UBE3A* mRNA levels could be manipulated pharmacologically. Future studies in which the expression levels of individual genes are reduced or that compare Dup15q neurons with isogenic control neurons may help delineate the precise roles of individual genes in this disorder.

## Availability of supporting data

The data set supporting the results of this article is available in the National Center for Biotechnology Information (NCBI) Sequence Read Archive (SRA), under the accession number SRP044749 (http://www.ncbi.nlm.nih.gov/sra/?term=srp044749).

## Abbreviations

ADORA2A: adenosine A2A receptor; ARX: aristaless related homebox; AS: Angelman syndrome; ASD: autism spectrum disorder; ATP10A: ATPase class V 10A; cAMP: cyclic adenosine monophosphate; ChIP: chromatin immunoprecipitation; CHRNA7: cholinergic receptor nicotinic alpha 7; CNQX: 6-cyano-7-nitroquinoxaline-2,3-dione; CNV: copy number variation; CYFIP1: cytoplasmic FMRP interacting protein; DLX2: distal-less homebox 2; DLX5: distal-less homeobox 5; DMSO: dimethyl sulfoxide; Dup15q: chromosome 15q11-q13.1 duplication; EB: embryoid body; EN1: engrailed 1; FISH: fluorescence in situ hybridization; FMRP: fragile X mental retardation protein; FOXG1: forkhead box G1; FPKM: fragments per kilobase gene model per million base pairs; GABA: gamma-aminobutyric acid; GAD65: glutamate decarboxylase; GAPDH: glyceraldehyde-3-phosphate dehydrogenase; HERC2: HECT and RLD domain contained E3 ubiquitin protein ligase 2; idic(15): isodicentric chromosome 15; int dup(15): interstitial duplication of 15q11-q13.1; iPSC: induced pluripotent stem cell; IPW: imprinted in Prader-Willi syndrome; ISL1: ISL LIM homeobox 1; KLF4: Kruppel-like factor 4; LIN28: lin28 homolog A; MAP2: microtubule associated protein 2; MEF: mouse embryonic fibroblasts; MYC: v-myc avian myelocytomatosis viral oncogene homolog; NCAM1: neural cell adhesion molecule 1; NCBI: National Center for Biotechnology Information; NIPA1/NIPA2: nonimprinted in Prader-Willi/Angelman syndrome region 1 and 2; NKX2.1: NK homeobox 2; NLGN1: neuroligin 1; OCT4: POU class 5 homeobox 1; OTX2: orthodenticle homeobox 2; PAX6: paired box 6; PSD95: postsynaptic density protein 95; PWS-IC: Prader-Willi syndrome imprinting center; qRT-PCR: quantitative reverse transcription polymerase chain reaction; RBFOX3: RNA binding protein, fox-1 homolog 3 (NEUN); RB1: retinoblastoma 1; SHANK1: SH3 and multiple ankyrin repeat domains 1; SNP: single nucleotide polymorphism; SNRPN: small nuclear ribonucleoprotein N; SOX2: SRY-box 2; SSEA4: state specific embryonic antigen 4; S100β: S100 calcium binding protein beta; TBR1: T-box brain 1; TUBGCP5: tubulin gamma complex associated protein 5; UBE3A: ubiquitin protein ligase E3A; VGlut1: vesicular glutamate transporter 1; YY-1: yin yang 1; ZFP42: zinc finger protein 42.

## Competing interests

All authors declare that they have no competing interests.

## Authors’ contributions

NDG and SJC conceived of and designed the study, collected and analyzed data, and wrote the manuscript. P-FC, JJF, and TMR collected and analyzed data. AMP analyzed data and provided critical revision of the manuscript. HG-D prepared sequencing libraries. JB collected and analyzed data and provided critical revision of the manuscript. ESL designed study, analyzed data, and wrote the manuscript. LTR acquired and processed patient samples and provided critical revision of the manuscript. BRG designed the study, analyzed data, and provided critical revision of the manuscript. ML conceived of and designed the study and provided critical revision of the manuscript. All authors read and approved the final manuscript.

## Supplementary Material

Additional file 1: Table S1 List of induced pluripotent stem cell (iPSC) lines used in this study, parental tissue source, and method of reprogramming used. iPSCs were generated from an Angelman syndrome (AS) individual (AS del 1–0), a normal individual (Nml 1–0), an individual with an interstitial duplication of the maternal 15q11-q13.1 allele (mat. int dup(15)-12 and mat. int dup(15)-02), an individual with an interstitial duplication of the paternal 15q11-q13.1 allele (pat. int dup(15)-04), and two individuals with an isodicentric chromosome 15 of maternal origin (Idic1-8 and IdicCB-09). The copy number of maternal or paternal 15q11-q13.1 in each cell line, the parental somatic cell source, and the reprogramming method used are indicated. Click here for file

Additional file 2: Figure S1 Characterization of pluripotency of Dup15q induced pluripotent stem cells (iPSCs). (A) Quantitative reverse transcription PCR (qRT-PCR) analysis for selected pluripotency genes using a TaqMan human pluripotency gene array with representative iPSC clones of each Dup15q genotype. H9 hESCs and iPSCs from a normal individual (Nml 1–0) are included as reference samples. (B) qRT-PCR analysis of day 16 embryoid bodies derived from representative clones of each Dup15q genotype using a TaqMan human pluripotency array. Click here for file

Additional file 3: Table S2 Methylation analysis of Dup15q induced pluripotent stem cells (iPSCs) at the Prader-Willi syndrome imprinting center (PWS-IC) (individual iPSC clones). DNA methylation at the PWS-IC was assayed by methylation specific quantitative PCR (qPCR) using genomic DNA from Dup15q iPSCs. The calculated percent methylation for each clone within a genotype is presented from one assay per clone. *The mat. int dup(15)-12 clone, while originally identified as having a normal methylation status of 54%, subsequently demonstrated the 66% methylation status indicative of the maternal interstitial duplication containing clones, suggesting that this clone was mosaic for the duplication. ** The mat. int dup(15)-18 clone was also shown to be a mixed clone by DNA fluorescence *in situ* hybridization (FISH) analysis using a probe for *SNRPN*. Click here for file

Additional file 4: Figure S2 Allele-specific reverse transcription PCR (RT-PCR) for paternally expressed imprinted in Prader-Willi syndrome (*IPW*) in patient fibroblasts, induced pluripotent stem cells (iPSCs), and iPSC-derived neurons. DNA sequencing of genomic DNA from either patient fibroblasts (mat. int dup(15), pat. int dup(15), and idic(15)) or iPSCs (idic(15) cord blood (IdicCB-09)) indicates the presence of a polymorphic single nucleotide polymorphism (SNP), rs691, in the last exon of the *imprinted in Prader*-*Willi syndrome* (*IPW*) gene (arrows). Sequencing of cDNA derived from patient fibroblasts, iPSCs, and 10-week-old iPSC-derived neurons demonstrates that expression of *IPW* in all samples is monoallelic. Expression of the same allele (as indicated by DNA sequencing traces containing the same nucleotide at rs691, (asterisks)) in fibroblasts, iPSCs, and iPSC-derived neurons suggests that the parental imprinting of *IPW* is maintained following reprogramming and differentiation of iPSCs into neural derivatives. We were unable to analyze cDNA from pat. int dup(15) fibroblasts and idic(15) cord blood due to a lack of sufficient patient cells for generating RNA. Click here for file

Additional file 5: Figure S3 Analysis of neural differentiation of Dup15q induced pluripotent stem cell (iPSC) clones using a custom neural differentiation quantitative PCR (qPCR) array. Each iPSC clone derived from Dup15q patient samples was assayed for neural differentiation capacity using a custom TaqMan neural differentiation qPCR array. iPSCs derived from individuals with a maternal interstitial duplication (A), paternal interstitial duplication (B), or idic(15) (fibroblast sample, C, and umbilical cord blood, D) were spontaneously differentiated via embryoid body formation for 16 days. Neural gene expression levels were analyzed relative to expression in AS del 1–0 day 16 embryoid bodies. AS del 1–0 is used as a calibrator sample since it has previously been published to readily generate mature neurons and astrocytes [18]. Embryoid bodies derived from H9 hESCs and normal iPSCs (Nml 1–0) were included in analysis for comparison purposes. The pluripotency genes *NANOG* and *ZFP42* were also included in analysis to assay downregulation of pluripotency genes during differentiation. Click here for file

Additional file 6: Figure S4 Characterization of Dup15q induced pluripotent stem cell (iPSC)-derived neural cultures. (A) Triplicate independently-derived 10-week-old neural cultures from each Dup15q cell line were analyzed by qRT-PCR for markers of neurons and glia (*βIII*-*tubulin*, *RBFOX3*, *TBR1*, and *S100β*) excitatory and inhibitory neurons (*VGLUT2* and *GAD1*), and forebrain and midbrain neurons (*FOXG1*, *PAX6*, *OTX2*, and *EN1*). Data are presented as mean expression relative to *GAPDH*. (B) Mean expression levels of each gene were compared between cell lines using one-way ANOVA followed by Tukey’s multiple comparison post hoc test. Pairwise comparisons having significant P values (P <0.05) for each gene are listed. Click here for file

Additional file 7: Figure S5 Expression of 15q11-q13.1 genes in Dup15q induced pluripotent stem cell (iPSCs) and iPSC-derived neurons. (A) qRT-PCR analysis of selected 15q11-q13.1 genes in iPSCs. iPSC lines are presented from left to right in order of increasing 15q11-q13.1 copy number. Genes are arranged along the x-axis in the order of their location within the 15q11-q13.1 region. (B) qRT-PCR analysis of selected 15q11-q13.1 genes in 10-week-old iPSC-derived neurons. Click here for file

Additional file 8: Figure S6 Analysis of *UBE3A* copy number in induced pluripotent stem cell (iPSCs). (A) The genomic copy number of *UBE3A* was analyzed by qPCR in each iPSC line using TaqMan Copy Number Assays. *UBE3A* copy number was calculated using RNase P as an endogenous reference. Error bars indicate standard error of the mean. (B) RNA FISH for *UBE3A* in iPSCs shows RNA expression from two (Nml 1–0), three (mat. int dup(15)-02 and mat. int dup(15)-12) or four (Idic1-8) *UBE3A* alleles. Arrows indicate positive FISH signal. Nuclei are labeled with DAPI (blue). Click here for file

Additional file 9: Table S3 mRNA-Seq data from AS (AS del1-0), normal (Nml 1-0), and idic(15) (Idic1-8) induced pluripotent stem cell (iPSC)-derived neurons. The tab titled “transcriptome data in neurons” shows the averaged fragments per kilobase pair per million mapped reads (FPKM) values for mRNA-Seq performed on two biological replicates of AS del1-0, Nml1-0, and Idic1-8 iPSC-derived neurons. The “AS vs. Normal” tab shows a scatter plot comparing the Log2 transformed FPKM+1 values for AS del1-0 and Nml1-0 iPSC-derived neurons. The “Idic(15) vs. AS” tab shows a scatter plot comparing the Log2 transformed FPKM+1 values for Idic1-8 and AS del1-0 iPSC-derived neurons. The “Idic(15) vs. Normal” tab shows a scatter plot comparing the Log2 transformed FPKM+1 values for Idic1-8 and Nml1-0 iPSC-derived neurons. The “Idic(15) vs. Idic(15)” tab shows a scatter plot comparing the Log2 transformed FPKM+1 values for the two biological replicates of Idic1-8 iPSC-derived neurons. Click here for file

Additional file 10: Table S4 Gene Ontology analysis of genes upregulated in idic(15) (Idic1-8) induced pluripotent stem cell (iPSC)-derived neurons. Genes upregulated in idic(15) iPSC-derived neurons at least twofold compared to normal were subject to analysis using DAVID (http://david.abcc.ncifcrf.gov). Gene ontology terms representing the function of the genes upregulated are shown along with the corresponding statistical analysis. Click here for file

Additional file 11: Table S5 Gene Ontology analysis of genes upregulated in AS del 1–0 induced pluripotent stem cell (iPSC)-derived neurons. Genes upregulated in AS iPSC-derived neurons at least twofold compared to normal were subject to analysis using DAVID (http://david.abcc.ncifcrf.gov). Gene ontology terms representing the function of the genes upregulated are shown along with the corresponding statistical analysis. Click here for file

Additional file 12: Table S6 Gene Ontology analysis of genes downregulated in idic(15) (Idic1-8) induced pluripotent stem cell (iPSC)-derived neurons. Genes downregulated in idic(15) iPSC-derived neurons at least twofold compared to normal were subject to analysis using DAVID (http://david.abcc.ncifcrf.gov). Gene ontology terms representing the function of the genes downregulated are shown along with the corresponding statistical analysis. Click here for file

Additional file 13: Table S7 Gene Ontology analysis of genes downregulated in AS del 1–0 induced pluripotent stem cell (iPSC)-derived neurons. Genes downregulated in AS iPSC-derived neurons at least twofold compared to normal were subject to analysis using DAVID (http://david.abcc.ncifcrf.gov). Gene ontology terms representing the function of the genes downregulated are shown along with the corresponding statistical analysis. Click here for file

Additional file 14: Figure S7 Analysis of autism candidate genes in maternal int dup(15) induced pluripotent stem cell (iPSC)-derived neurons. Differential gene expression of select autism candidate genes and genes implicated in seizure disorders were analyzed by qRT-PCR in 10-week-old normal (Nml 1–0), maternal int dup(15) (mat int dup(15)-02), and idic(15) (Idic1-8) iPSC-derived neurons. *P* values: *P ≤0.1, **P ≤0.05, ***P ≤0.01. Click here for file

Additional file 15: Figure S8 Off-target effects of mithramycin treatment in idic(15) induced pluripotent stem cell (iPSC)-derived neurons. qRT-PCR analysis of *SNRPN*, *GABRB3*, and *CYFIP1* expression in 10-week-old idic(15) (Idic1-8) iPSC-derived neurons following 72 hours of treatment with mithramycin or DMSO. *P* values: *P ≤0.1, **P ≤0.05, ***P ≤0.01. Click here for file

Additional file 16: Figure S9 Chromatin immunoprecipitation (ChIP) analysis of Sp1 and YY-1 binding at the *UBE3A* promoter. (A) ChIP-qPCR assays were performed on idic(15) (Idic1-8) iPSC-derived neurons using two different antibodies against Sp1. With anti-Sp1 antibody #1 (Santa Cruz Biotechnology, Inc.), we were unable to detect significant enrichment over background signal (IgG) suggesting that this antibody does not work in our hands. Using anti-Sp1 antibody #2 (Cell Signaling Technologies), we detected low levels of enrichment at two sites at *UBE3A* (one within exon 1 and one upstream of exon 1), however, when compared to enrichment at the Sp1 promoter and the *DHFR* promoter – two positive control locations, we concluded that binding of Sp1 to *UBE3A* is not significant. (B) ChIP-qPCR assays were performed on idic(15) (Idic1-8) iPSC-derived neurons to analyze binding of YY-1 to two locations at *UBE3A*, one within exon 1 and one upstream of exon 1. Data are presented as the mean enrichment from triplicate ChIP experiments plus or minus the standard error of the mean. Click here for file
