# Supplementary figures and images for: Gene expression analysis of human induced pluripotent stem cell-derived neurons carrying copy number variants of chromosome 15q11-q13.1
Source: Mol Autism. 2014 Aug 20;5:44. doi: 10.1186/2040-2392-5-44 (PMC4332023; doi:10.1186/2040-2392-5-44)

**A**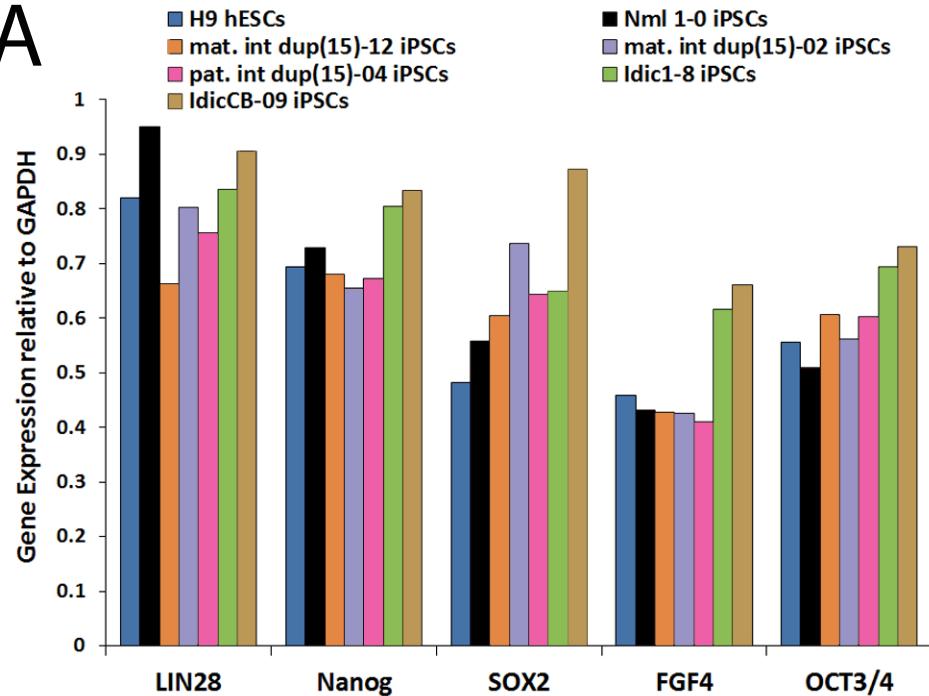**B**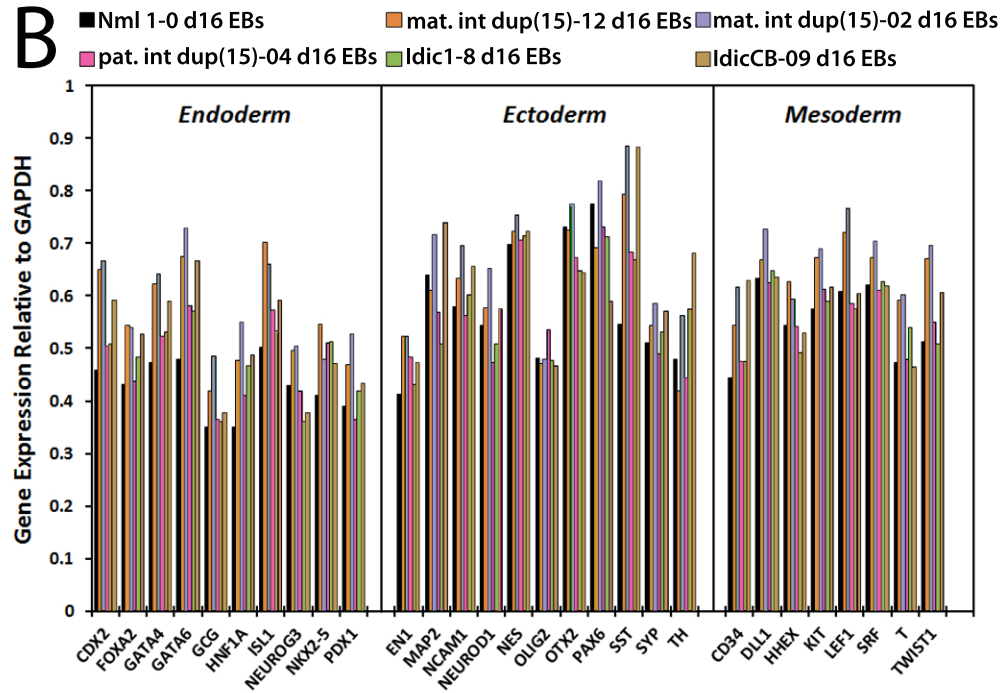

Supplement: Additional file 2: Figure S1 — Characterization of pluripotency of Dup15q induced pluripotent stem cells (iPSCs). (A) Quantitative reverse transcription PCR (qRT-PCR) analysis for selected pluripotency genes using a TaqMan human pluripotency gene array with representative iPSC clones of each Dup15q genotype. H9 hESCs and iPSCs from a normal individual (Nml 1–0) are included as reference samples. (B) qRT-PCR analysis of day 16 embryoid bodies derived from representative clones of each Dup15q genotype using a TaqMan human pluripotency array. [file 2040-2392-5-44-S2.pdf]

A

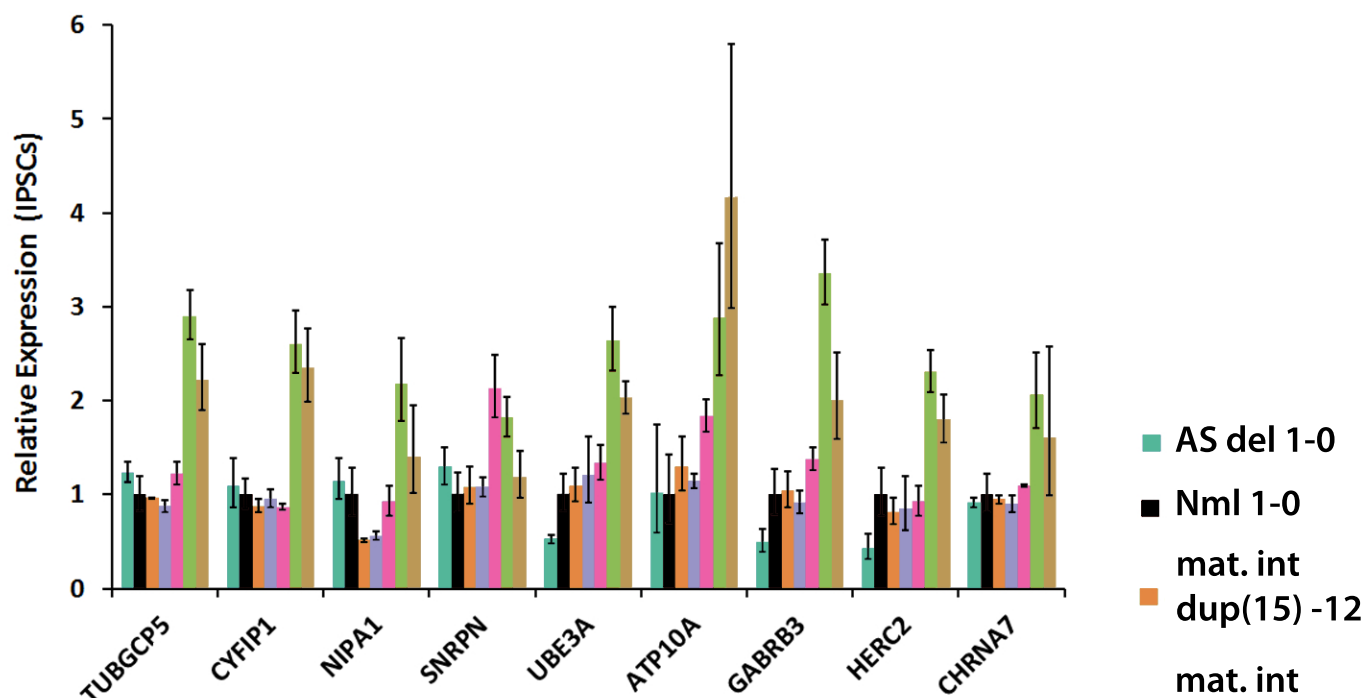

B

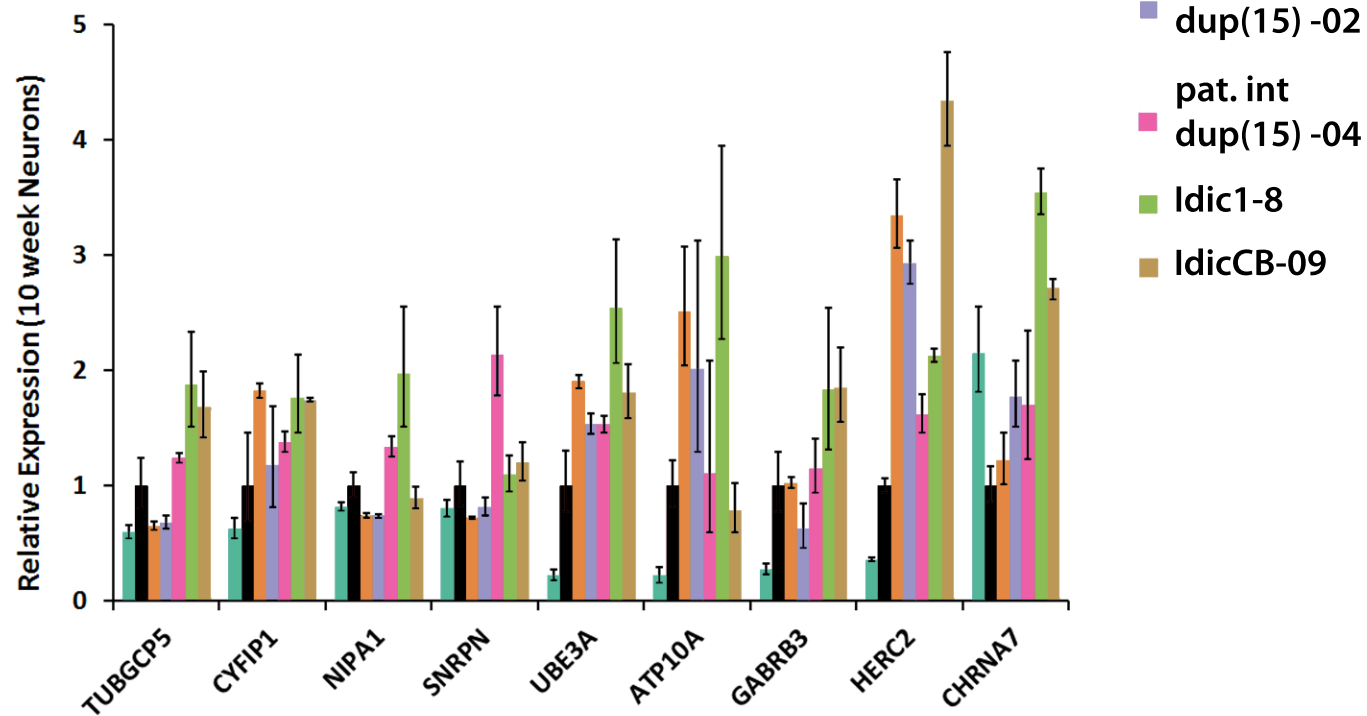

Supplement: Additional file 7: Figure S5 — Expression of 15q11-q13.1 genes in Dup15q induced pluripotent stem cell (iPSCs) and iPSC-derived neurons. (A) qRT-PCR analysis of selected 15q11-q13.1 genes in iPSCs. iPSC lines are presented from left to right in order of increasing 15q11-q13.1 copy number. Genes are arranged along the x-axis in the order of their location within the 15q11-q13.1 region. (B) qRT-PCR analysis of selected 15q11-q13.1 genes in 10-week-old iPSC-derived neurons. [file 2040-2392-5-44-S7.pdf]

**A**

AS del 1-0      Nml 1-0      mat.int dup(15)-12  
mat.int dup(15)-02      pat.int dup(15)-04      Idic1-8

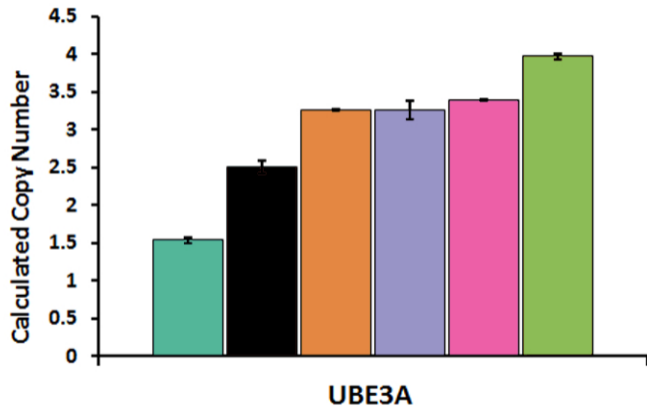**B**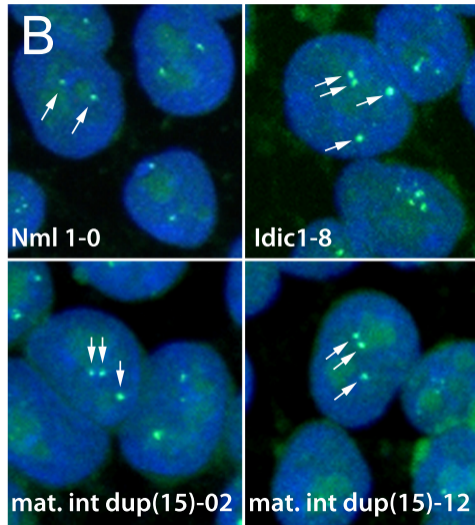

Supplement: Additional file 8: Figure S6 — Analysis of UBE3A copy number in induced pluripotent stem cell (iPSCs). (A) The genomic copy number of UBE3A was analyzed by qPCR in each iPSC line using TaqMan Copy Number Assays. UBE3A copy number was calculated using RNase P as an endogenous reference. Error bars indicate standard error of the mean. (B) RNA FISH for UBE3A in iPSCs shows RNA expression from two (Nml 1–0), three (mat. int dup(15)-02 and mat. int dup(15)-12) or four (Idic1-8) UBE3A alleles. Arrows indicate positive FISH signal. Nuclei are labeled with DAPI (blue). [file 2040-2392-5-44-S8.pdf]

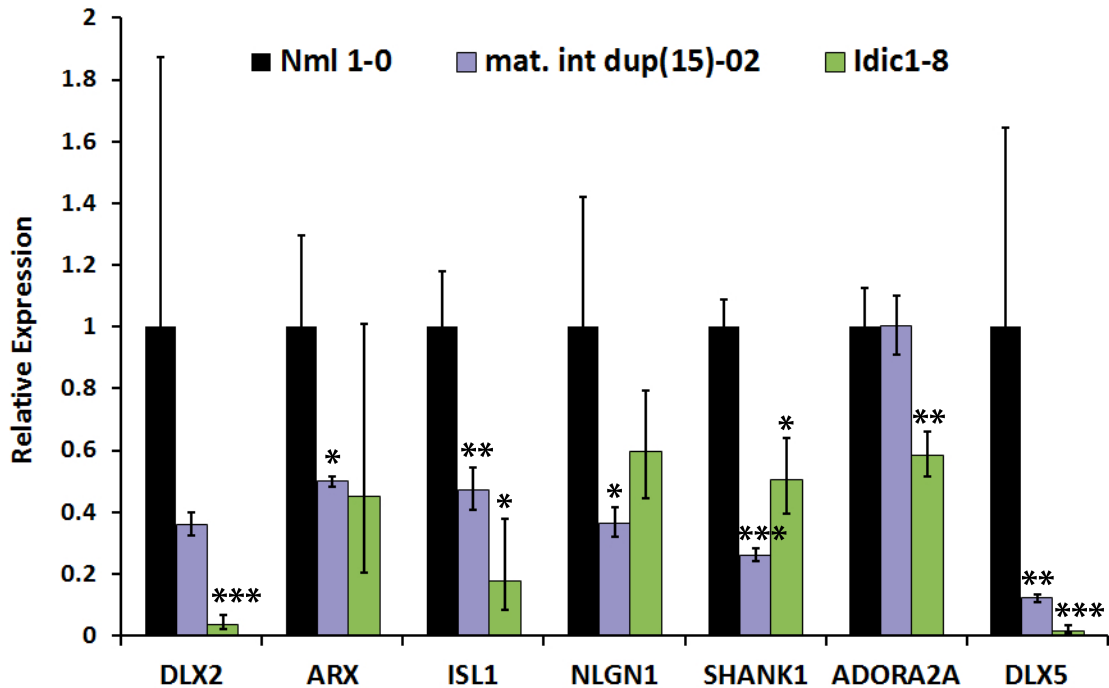

Supplement: Additional file 14: Figure S7 — Analysis of autism candidate genes in maternal int dup(15) induced pluripotent stem cell (iPSC)-derived neurons. Differential gene expression of select autism candidate genes and genes implicated in seizure disorders were analyzed by qRT-PCR in 10-week-old normal (Nml 1–0), maternal int dup(15) (mat int dup(15)-02), and idic(15) (Idic1-8) iPSC-derived neurons. P values: *P ≤0.1, **P ≤0.05, ***P ≤0.01. [file 2040-2392-5-44-S14.pdf]

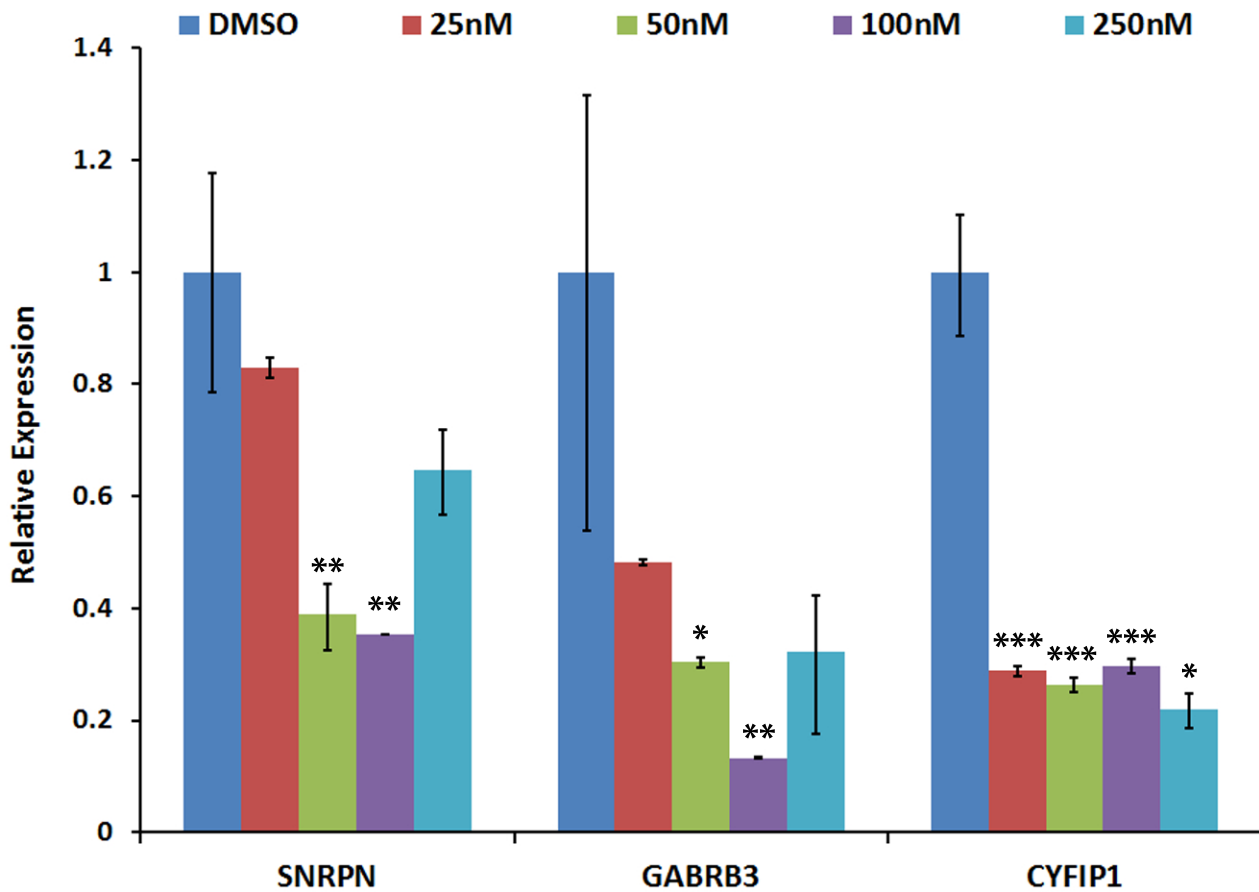

Supplement: Additional file 15: Figure S8 — Off-target effects of mithramycin treatment in idic(15) induced pluripotent stem cell (iPSC)-derived neurons. qRT-PCR analysis of SNRPN, GABRB3, and CYFIP1 expression in 10-week-old idic(15) (Idic1-8) iPSC-derived neurons following 72 hours of treatment with mithramycin or DMSO. P values: *P ≤0.1, **P ≤0.05, ***P ≤0.01. [file 2040-2392-5-44-S15.pdf]

A

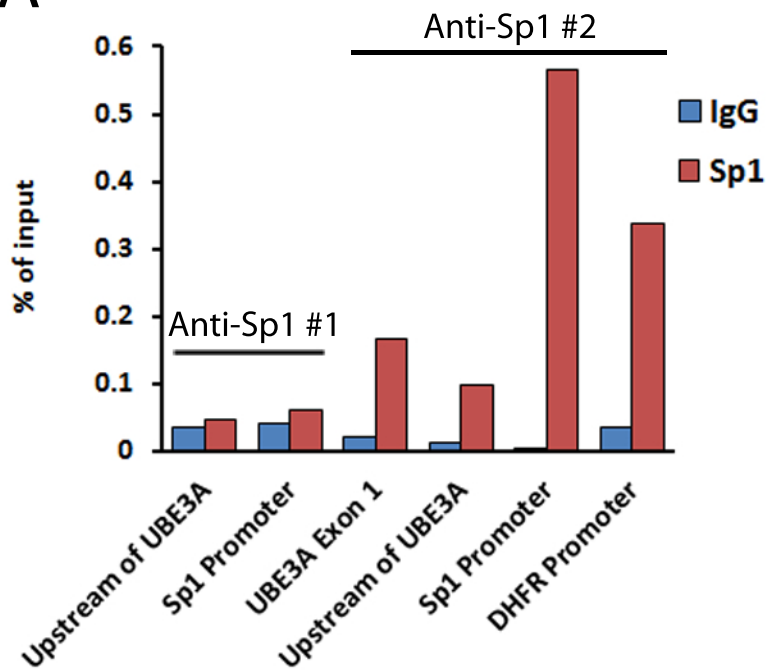

B

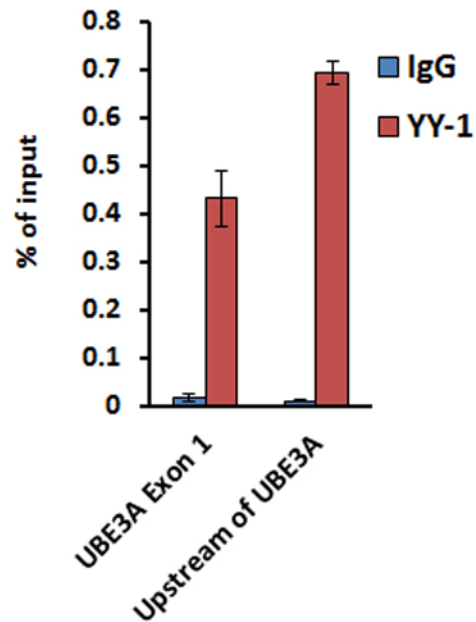

Supplement: Additional file 16: Figure S9 — Chromatin immunoprecipitation (ChIP) analysis of Sp1 and YY-1 binding at the UBE3A promoter. (A) ChIP-qPCR assays were performed on idic(15) (Idic1-8) iPSC-derived neurons using two different antibodies against Sp1. With anti-Sp1 antibody #1 (Santa Cruz Biotechnology, Inc.), we were unable to detect significant enrichment over background signal (IgG) suggesting that this antibody does not work in our hands. Using anti-Sp1 antibody #2 (Cell Signaling Technologies), we detected low levels of enrichment at two sites at UBE3A (one within exon 1 and one upstream of exon 1), however, when compared to enrichment at the Sp1 promoter and the DHFR promoter – two positive control locations, we concluded that binding of Sp1 to UBE3A is not significant. (B) ChIP-qPCR assays were performed on idic(15) (Idic1-8) iPSC-derived neurons to analyze binding of YY-1 to two locations at UBE3A, one within exon 1 and one upstream of exon 1. Data are presented as the mean enrichment from triplicate ChIP experiments plus or minus the standard error of the mean. [file 2040-2392-5-44-S16.pdf]
