# Supplementary material for: Gene expression analysis of human induced pluripotent stem cell-derived neurons carrying copy number variants of chromosome 15q11-q13.1
Source: Mol Autism. 2014 Aug 20;5:44. doi: 10.1186/2040-2392-5-44 (PMC4332023; doi:10.1186/2040-2392-5-44)
Supplement: Additional file 4: Figure S2 — Allele-specific reverse transcription PCR (RT-PCR) for paternally expressed imprinted in Prader-Willi syndrome (IPW) in patient fibroblasts, induced pluripotent stem cells (iPSCs), and iPSC-derived neurons. DNA sequencing of genomic DNA from either patient fibroblasts (mat. int dup(15), pat. int dup(15), and idic(15)) or iPSCs (idic(15) cord blood (IdicCB-09)) indicates the presence of a polymorphic single nucleotide polymorphism (SNP), rs691, in the last exon of the imprinted in Prader-Willi syndrome (IPW) gene (arrows). Sequencing of cDNA derived from patient fibroblasts, iPSCs, and 10-week-old iPSC-derived neurons demonstrates that expression of IPW in all samples is monoallelic. Expression of the same allele (as indicated by DNA sequencing traces containing the same nucleotide at rs691, (asterisks)) in fibroblasts, iPSCs, and iPSC-derived neurons suggests that the parental imprinting of IPW is maintained following reprogramming and differentiation of iPSCs into neural derivatives. We were unable to analyze cDNA from pat. int dup(15) fibroblasts and idic(15) cord blood due to a lack of sufficient patient cells for generating RNA. [file 2040-2392-5-44-S4.pdf]

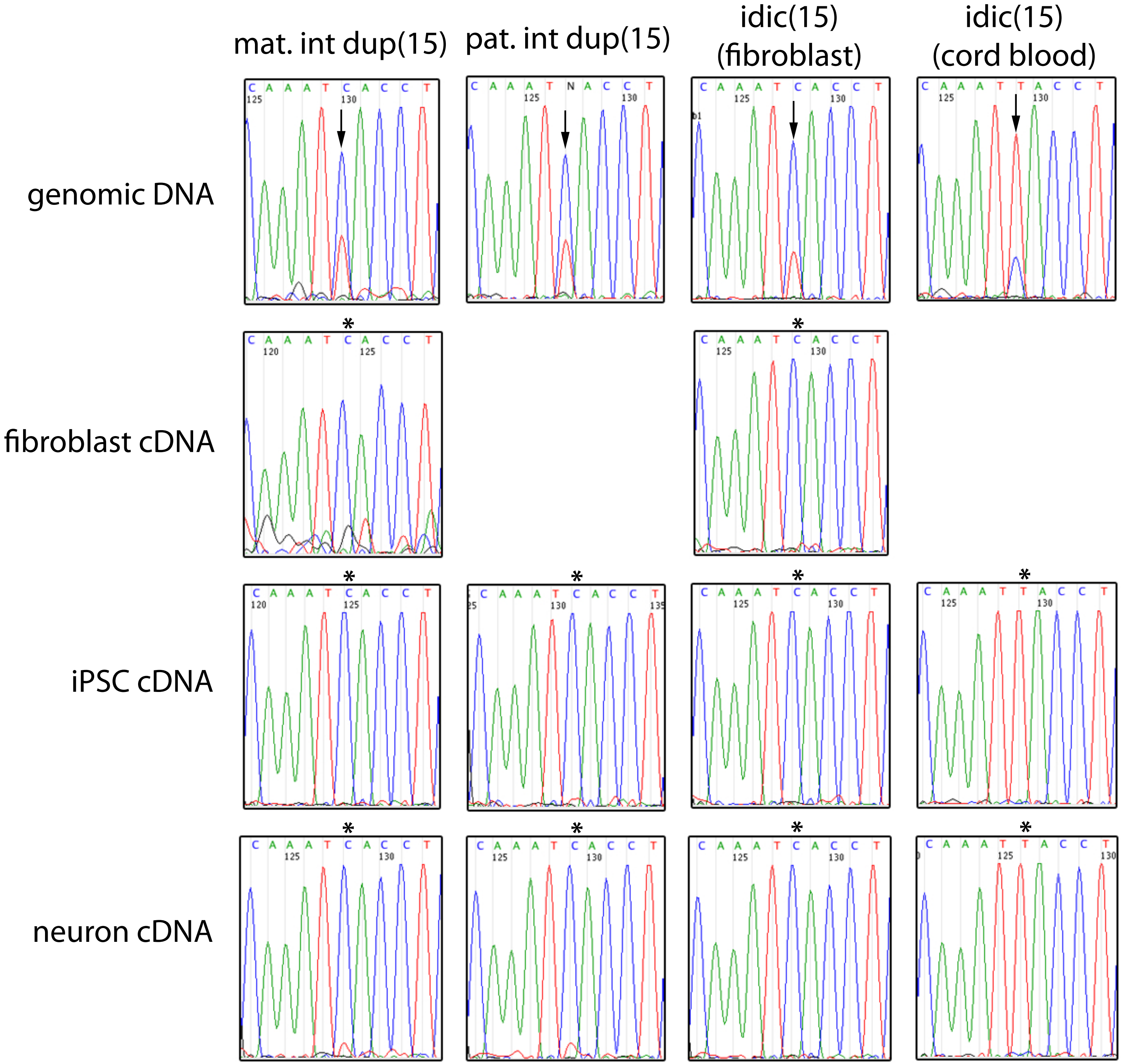

mat. int dup(15)
pat. int dup(15)
idic(15) (fibroblast)
idic(15) (cord blood)
genomic DNA
fibroblast cDNA
iPSC cDNA
neuron cDNA
