# Supplementary material for: Gene expression analysis of human induced pluripotent stem cell-derived neurons carrying copy number variants of chromosome 15q11-q13.1
Source: Mol Autism. 2014 Aug 20;5:44. doi: 10.1186/2040-2392-5-44 (PMC4332023; doi:10.1186/2040-2392-5-44)
Supplement: Additional file 5: Figure S3 — Analysis of neural differentiation of Dup15q induced pluripotent stem cell (iPSC) clones using a custom neural differentiation quantitative PCR (qPCR) array. Each iPSC clone derived from Dup15q patient samples was assayed for neural differentiation capacity using a custom TaqMan neural differentiation qPCR array. iPSCs derived from individuals with a maternal interstitial duplication (A), paternal interstitial duplication (B), or idic(15) (fibroblast sample, C, and umbilical cord blood, D) were spontaneously differentiated via embryoid body formation for 16 days. Neural gene expression levels were analyzed relative to expression in AS del 1–0 day 16 embryoid bodies. AS del 1–0 is used as a calibrator sample since it has previously been published to readily generate mature neurons and astrocytes [18]. Embryoid bodies derived from H9 hESCs and normal iPSCs (Nml 1–0) were included in analysis for comparison purposes. The pluripotency genes NANOG and ZFP42 were also included in analysis to assay downregulation of pluripotency genes during differentiation. [file 2040-2392-5-44-S5.pdf]

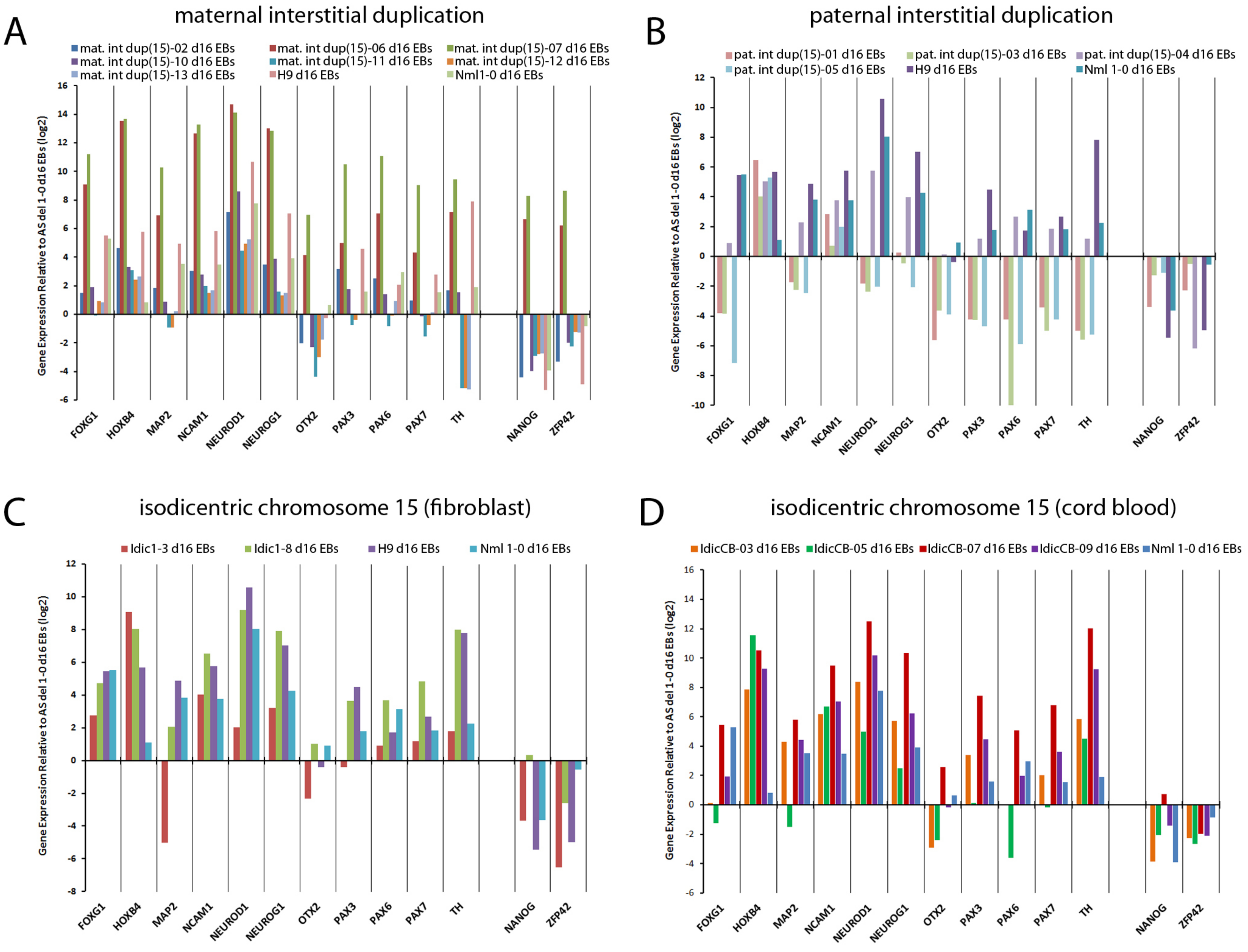
A
maternal interstitial duplication
mat. int dup(15)-02 d16 EBs
mat. int dup(15)-06 d16 EBs
mat. int dup(15)-07 d16 EBs
mat. int dup(15)-10 d16 EBs
mat. int dup(15)-11 d16 EBs
mat. int dup(15)-12 d16 EBs
mat. int dup(15)-13 d16 EBs
H9 d16 EBs
Nml1-0 d16 EBs
Gene Expression Relative to AS del 1-0 d16 EBs (log2)
FOXG1
HOXB4
MAP2
NCAM1
NEUROD1
NEUROG1
OTX2
PAX3
PAX6
PAX7
TH
NANOG
ZFP42
B
paternal interstitial duplication
pat. int dup(15)-01 d16 EBs
pat. int dup(15)-03 d16 EBs
pat. int dup(15)-04 d16 EBs
pat. int dup(15)-05 d16 EBs
H9 d16 EBs
Nml 1-0 d16 EBs
C
isodicentric chromosome 15 (fibroblast)
Idic1-3 d16 EBs
Idic1-8 d16 EBs
H9 d16 EBs
Nml 1-0 d16 EBs
D
isodicentric chromosome 15 (cord blood)
IdicCB-03 d16 EBs
IdicCB-05 d16 EBs
IdicCB-07 d16 EBs
IdicCB-09 d16 EBs
Nml 1-0 d16 EBs
