# Supplementary material for: Gene expression analysis of human induced pluripotent stem cell-derived neurons carrying copy number variants of chromosome 15q11-q13.1
Source: Mol Autism. 2014 Aug 20;5:44. doi: 10.1186/2040-2392-5-44 (PMC4332023; doi:10.1186/2040-2392-5-44)
Supplement: Additional file 6: Figure S4 — Characterization of Dup15q induced pluripotent stem cell (iPSC)-derived neural cultures. (A) Triplicate independently-derived 10-week-old neural cultures from each Dup15q cell line were analyzed by qRT-PCR for markers of neurons and glia (βIII-tubulin, RBFOX3, TBR1, and S100β) excitatory and inhibitory neurons (VGLUT2 and GAD1), and forebrain and midbrain neurons (FOXG1, PAX6, OTX2, and EN1). Data are presented as mean expression relative to GAPDH. (B) Mean expression levels of each gene were compared between cell lines using one-way ANOVA followed by Tukey’s multiple comparison post hoc test. Pairwise comparisons having significant P values (P <0.05) for each gene are listed. [file 2040-2392-5-44-S6.pdf]

A

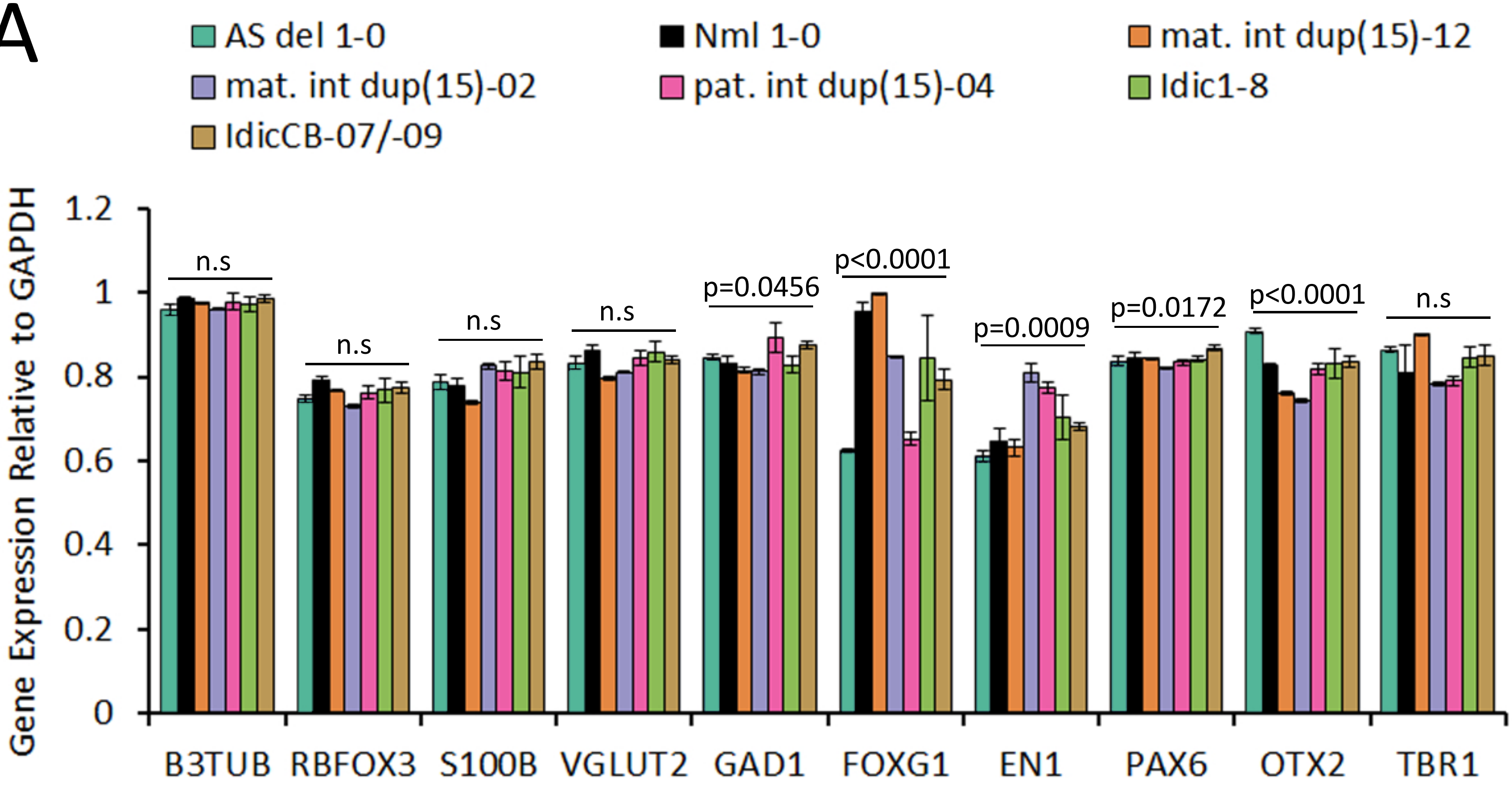

B

P<0.05 by Tukey 's Multiple Comparison Test

GAD1

No pairwise comparisons had P<0.05

FOXG1

mat. int dup(15)-12 *vs* AS del 1-0
mat. int dup(15)-12 *vs* pat. int dup(15)-04
mat. int dup(15)-12 *vs* IdicCB-07/09
mat. int dup(15)-02 *vs* AS del 1-0
Idic1-8 *vs* AS del 1-0
AS del 1-0 *vs* Nml 1-0
pat. int dup(15)-04 *vs* Nml 1-0

EN1

mat. int dup(15)-12 *vs* mat. int dup(15)-02
mat. int dup(15)-12 *vs* pat. int dup(15)-04
mat. int dup(15)-02 *vs* AS del 1-0
mat. int dup(15)-02 *vs* Nml 1-0
AS del 1-0 *vs* pat. int dup(15)-04

PAX6

mat. int dup(15)-02 *vs* IdicCB-07/09

OTX2

mat. int dup(15)-12 *vs* AS del 1-0
mat. int dup(15)-12 *vs* IdicCB-07/09
mat. int dup(15)-02 *vs* Idic1-8
mat. int dup(15)-02 *vs* AS del 1-0
mat. int dup(15)-02 *vs* Nml 1-0
mat. int dup(15)-02 *vs* IdicCB-07/09
Idic1-8 *vs* AS del 1-0
AS del 1-0 *vs* pat. int dup(15)-04
AS del 1-0 *vs* Nml 1-0
